# Efficacy and safety of transdermal electrical stimulation in patients with nonarteritic anterior ischemic optic neuropathy

**DOI:** 10.1186/s42234-023-00125-2

**Published:** 2023-10-25

**Authors:** Gen Miura, Tadami Fujiwara, Yoshihito Ozawa, Yuki Shiko, Yohei Kawasaki, Tomohiro Nizawa, Tomoaki Tatsumi, Takuji Kurimoto, Sotaro Mori, Makoto Nakamura, Hideki Hanaoka, Takayuki Baba, Shuichi Yamamoto

**Affiliations:** 1 Department of Ophthalmology and Visual Science, Chiba University Graduate School of Medicine, Inohana 1-8-1, Chuo-Ku, Chiba, 260-8670 Japan; 2 Clinical Research Centre, Chiba University Hospital, Chiba, Japan; 3 Department of Surgery, Division of Ophthalmology, Kobe University Graduate School of Medicine, 7-5-1 Kusunoki-Cho, Chuo-Ku, Kobe, 650-0017 Japan

**Keywords:** Nonarteritic anterior ischemic optic neuropathy, NAION, Electrical stimulation, Skin electrode, TES

## Abstract

**Background:**

No effective treatment for NAION with strong evidence has been established till date. The aim of this investigator-led, prospective, non-randomized, open-label, uncontrolled multi-center exploratory clinical trial is to evaluate the efficacy and safety of transdermal electrical stimulation (TdES) using skin electrodes in patients with NAION.

**Methods:**

Five patients with monocular NAION underwent TdES (10-ms biphasic pulses, 1.0 mA, 20 Hz, 30 min) of the affected eye six times at 2-week intervals. The primary endpoint was the logarithm of the mini-mum angle of resolution (logMAR) visual acuity at 12 weeks compared with 0 weeks. The secondary endpoints were changes in the best-corrected logMAR visual acuity, Early Treatment of Diabetic Retinopathy Study (ETDRS) visual acuity, and mean deviation (MD) of the Humphrey field analyzer (HFA) 10–2 and HFA Esterman test scores. Additionally, the safety of TdES was evaluated.

**Results:**

LogMAR visual acuity improved by ≥ 0.1 in two eyes, and ETDRS visual acu-ity improved by ≥ 5 characters in one eye. The mean change in logMAR visual acuity from week 0 showed an increasing trend. The mean MD of HFA 10–2 showed no obvious change, while HFA Esterman score improved in four eyes. All patients completed the study according to the protocol, and no treatment-related adverse events were observed.

**Conclusions:**

TdES treatment may have improved visual acuity and visual field in some patients. Further sham-controlled study in larger cohort is needed on its effectiveness.

**Trial registration:**

UMIN, UMIN000036220. Registered 15 March, 2019, https://center6.umin.ac.jp/cgi-open-bin/ctr/ctr_view.cgi?recptno=R000041261.

**Supplementary Information:**

The online version contains supplementary material available at 10.1186/s42234-023-00125-2.

## Background

Improvement in visual function by electrical stimulation therapy has been reported in patients with retinitis pigmentosa (RP) (Schatz et al. 2017), dry age-related macular degeneration (Shinoda et al. 2008), glaucoma (Ota et al. 2018), and retinal artery occlusion (Naycheva et al. 2013; Miura et al. 2023).

Previous studies have shown that the improvement in visual function by ocular electrical stimulation is related to the improvement in the survival rate and functional activation of retinal ganglion cells (RGC) due to the activation of insulin-like growth factor-1 (Morimoto et al. 2005), brain derived neurotrophic factor (Sato et al. 2008), ciliary neurotrophic factor (Ni et al. 2009) and fibroblast growth factor-2 (Ciavatta et al. 2009).

**Fig. 1:**
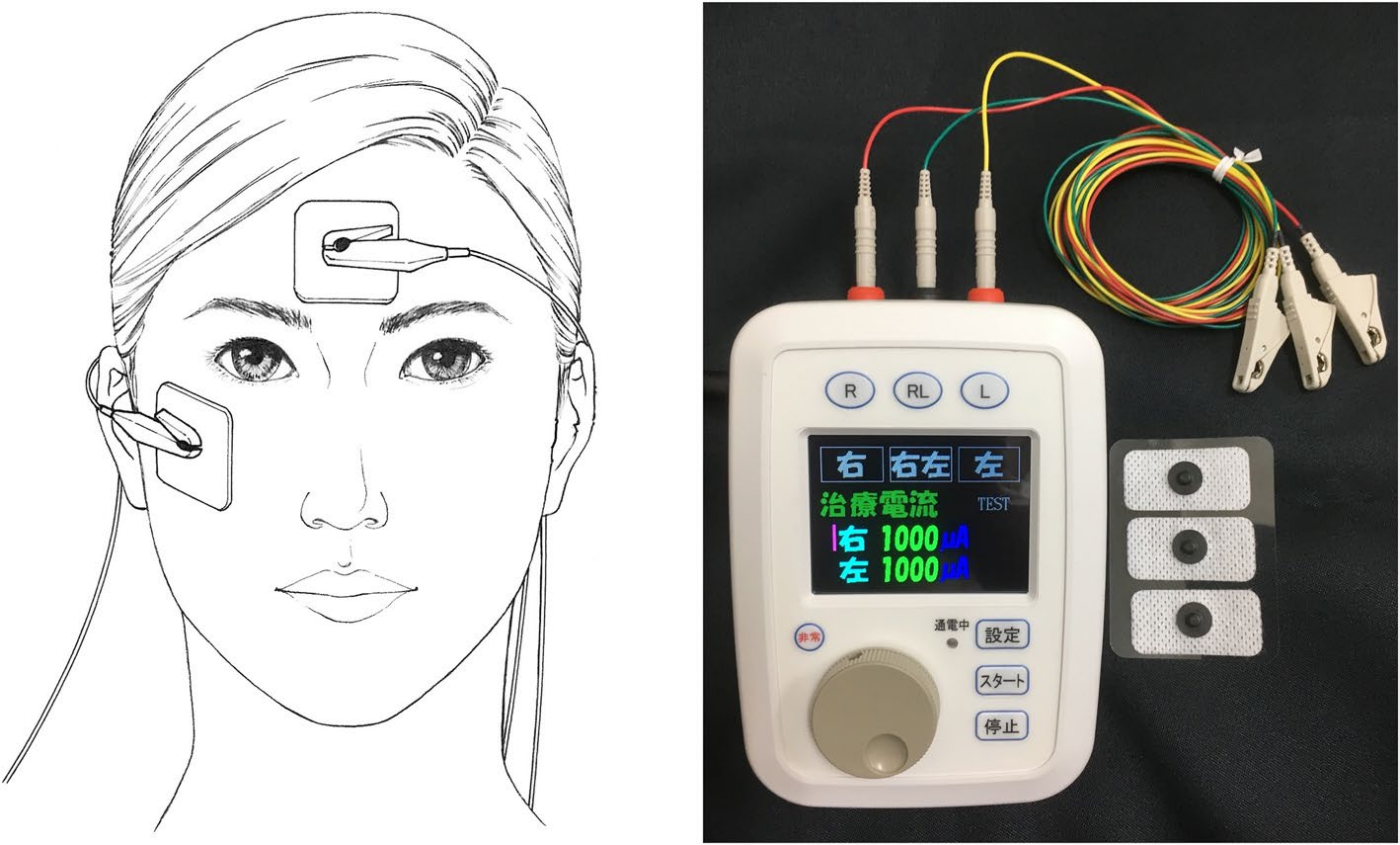
The prototype equipment for transdermal electrical stimulation. While one electrode was placed on the lower eyelid lateral to the midline of the affected eye (right eye in this figure), the other was placed at the center of the forehead

Most clinical studies on ocular electrical stimulation have used corneal electrodes. In clinical trials using transcorneal stimulation, mild superficial keratitis {Inomata, 2007 #232} {Morimoto, 2006 #46} and foreign-body sensation {Naycheva, 2013 #275} were reported with contact lens-type electrodes. In addition, foreign body sensation, conjunctival Irritation {Schatz, 2011 #280} and dry eye symptoms (31 of 52 subjects) {Schatz, 2017 #273} were reported with Dawson, Trick and Litzkow fiber electrodes. Therefore, we, jointly with Mayo Co. Ltd, developed a transdermal electrical stimulation (TdES) device that uses skin electrodes (Fig. 1).

Previously, we performed a clinical trial to evaluate the effects of electrical stimulation therapy using the TdES device in patients with RP. In that study, we confirmed that TdES does not cause any complications, and the visual acuity and mean deviation (MD) of the Humphrey field analyzer (HFA)10–2 were significantly improved after TdES (Miura et al. 2019). Based on these results, a larger, longer-term, multicenter, next-phase clinical trial is currently underway for patients with RP (Miura et al. 2022).

The pathogenesis of nonarteritic anterior ischemic optic neuropathy (NAION) is believed to be associated with impaired blood flow in the posterior ciliary artery, which results in sudden ischemia of the optic nerve head and loss of visual acuity and visual field.

NAION mainly occurs in relatively elderly patients, and the frequency of onset is thought to be approximately 2–10 per 100,000 people aged ≥ 50 years.

In most cases, the disease is unilateral, with unpredictable visual impairment and narrowing of the visual field, which is typical of horizontal visual field defects, but may also indicate central visual field defects. Optic nerve atrophy develops 4–8 weeks after onset.

Oral aspirin, levodopa, brimonidine, and prednisone; optic nerve-sheath decompression; and intravitreal injection of erythropoietin, triamcinolone, bevacizumab, and ranibizumab have been reported as treatment options for NAION (Lantos et al. 2022). However, no effective treatment for NAION with strong evidence has been established till date.

NAION is associated with the loss of RGC. Since ocular electrical stimulation improves the survival rate and functional activation of ganglion cells in the inner retina, it is considered effective for optic neuropathies such as NAION. However, few studies have used electrical stimulation for NAION. Fujikado et al. performed TES for patients with NAION and traumatic optic neuropathy and reported an improvement in visual acuity (Fujikado et al. 2006). While they performed electrical stimulation using a corneal electrode, to the best of our knowledge, no study has reported TdES using skin electrodes in patients with NAION. Thus, the purpose of this exploratory clinical trial was to evaluate the efficacy and safety of TdES in patients with NAION.

## Methods

This exploratory clinical trial was an investigator-led, prospective, non-randomized, open-label, uncontrolled multi-center trial conducted at Chiba University Hospital, Kobe University Hospital, and Nagoya City University Hospital in Japan. This 12-week trial consisted of six TdES treatments in patients with NAION. TdES was applied every 2 weeks for 6 weeks, as in our previous trial conducted in patients with RP (Miura et al. 2019). This trial was registered in the UMIN Clinical Trials Registry (UMIN000036220) on March 15, 2019, https://center6.umin.ac.jp/cgi-open-bin/ctr/ctr_view.cgi?recptno=R000041261.

The diagnosis of NAION was made based on a history of sudden monocular visual-acuity and visual-field loss, optic disc edema, and segmentally delayed optic disc filling and delayed choroid filling around the optic disc in the early stage and fluorescence leakage from the optic disc in the later stage on fluorescent fundus angiography. Intraorbital and intracranial diseases were ruled out using contrast-enhanced magnetic resonance imaging. In addition, giant-cell arteritis was ruled out through laboratory tests such as erythrocyte sedimentation rate (ESR), complete blood count, and C-reactive protein level in all cases.

Only patients with NAION onset ≥ 6 months previously were enrolled, as visual function may improve spontaneously > 180 days after onset (Hayreh 2014).

All patients underwent TdES consisting of 10-ms biphasic pulses of 1.0 mA at 20 Hz for 30 min only on the affected eye. One electrode was placed on the lower eyelid lateral to the midline of the affected eye and the other was placed at the center of the forehead. Electrical pulses were obtained from the same equipment jointly developed with Mayo Co., Ltd. (Aichi, Japan), which was used in a previous clinical trial.

### Eligibility criteria

Eligible patients were those who met all of the following inclusion criteria and did not have any listed exclusion criteria.

### Inclusion criteria

1. Clinically diagnosed with NAION and aged ≥ 20 years and ≤ 80 years.
2. Patients with NAION with persistent symptoms for > 6 months after onset.
3. Decimal visual acuity from hand motion to 0.7.
4. Patients who provided written informed consent for participation in this trial.
5. Regular hospital visits every 2 weeks for 12 weeks.

### Exclusion criteria

1. Patients with ESR > 20 mm/h and C-reactive protein level > 10 mg/L
2. History of allergy to mydriatic agents and ocular-surface anesthetics.
3. Presence of vitreous macular traction syndrome, macular edema, epiretinal membrane, myopia with posterior staphyloma, diabetic retinopathy, conjunctival inflammation, ocular infection, severe dry eye, grade 3 or higher Emery–Little grade cataract, and posterior capsule opacification.
4. Patients with a history or complications of malignant tumors. However, patients with a history who had not relapsed for > 5 years were enrolled.
5. Patients diagnosed with dementia or mental disorders and receiving treatment.
6. Patients with diabetes mellitus (HbA1c [National Glycohemoglobin Standardization Program] > 10.0%).
7. Patients with hypertension (systolic blood pressure ≥ 180 mmHg and/or diastolic blood pressure ≥ 110 mmHg) that was difficult to control with oral treatment.
8. Patients with any of the following on a screening blood sampling test.
  Aspartate aminotransferase and alanine aminotransferase levels > three times the upper limit of the facility standard value
  Serum creatinine level > 1.5 times the upper limit of the facility standard value
9. Patients taking ethambutol hydrochloride and/or amiodarone hydrochloride.
10. Patients who were pregnant, breastfeeding, or planned to be pregnant during the trial period.
11. Patients participating in other clinical trials.
12. Patients under investigational responsibility (shared) that the doctors judged inappropriate for participation in this trial.

The primary endpoint was the logarithm of the minimum angle of resolution (logMAR) visual acuity at 12 weeks compared with 0 weeks, and the secondary endpoints were the changes in the logMAR visual acuity, Early Treatment of Diabetic Retinopathy Study (ETDRS) visual acuity, MD of the HFA 10–2 and HFA monocular Esterman test scores. In addition, the incidence of adverse events (type, frequency, and severity) was investigated for safety evaluation.

Electrical stimulation was performed every 2 weeks for six sessions, and patients were assessed before beginning the TdES (baseline), 1 h after the end of treatment, and at 2 weeks after the TdES.

The TdES treatment and examination schedule for this trial are shown in Table 1. The study protocol was approved by the Institutional Review Boards of Chiba, Kobe, and Nagoya City University Hospitals. Written informed consent was obtained from all patients before enrollment.

**Table 1:** Treatment and examination schedule

	**Screening**	**0 week**	**2 weeks**	**4 weeks**	**6 weeks**	**8 weeks**	**10 weeks**	**12 weeks**	**Drop out**
	Visit 1–2	Visit 3	Visit 4	Visit 5	Visit 6	Visit 7	Visit 8	Visit 9	
IC	**○**								
TdES		**①**	**②**	**③**	**④**	**⑤**	**⑥**		
Blood pressure	**○**								
Blood sampling	**○**								
logMAR VA	**○**	**○**	**○**	**○**	**○**	**○**	**○**	**○**	**○**
ETDRS VA	**○**	**○**						**○**	**○**
HFA	**○**							**○**	**○**
Slit	**○**			**○**				**○**	**○**

*Abbreviations*: *IC* Informed consent, *TdES* Transdermal electrical stimulation, *VA* Visual acuity, *logMAR* Logarithm of the minimum angle of resolution, *ETDRS* Early Treatment of Diabetic Retinopathy Study, *HFA* Humphrey field analyzer, *Slit* Slit lamp examination

All patients underwent ophthalmic examinations, including measurement of best-corrected visual acuity (BCVA) and intraocular pressure. In addition, slit-lamp and indirect ophthalmoscopic examinations were performed. BCVA was measured monocularly using a standard Japanese Landolt ring chart.

Decimal visual acuity was converted to logMAR units for statistical analysis. For logMAR BCVA, finger counting and hand movement were defined as 1.8, light perception as 1.9, and loss of light perception as 2.0.

BCVA was also assessed using the ETDRS chart (CSV-1000LanC VectorVision, Ohio, USA) at a distance of 2.5 m. The luminance for the tests was 85 cd/m^2^, which is the luminance recommended for vision testing by the United States National Academy of Sciences, and adopted by the FDA as the required testing light level for clinical trials.

The MDs of the retinal sensitivity and monocular Esterman test score were determined using the Humphrey Visual Field Analyzer III (Model 850; Carl Zeiss Meditec, Inc., Dublin, CA, USA) with the Swedish Interactive Threshold Algorithm Standard 10–2 protocol and the monocular Esterman test.

The electrical stimulation settings, testing equipment, and measurement methods presented here overlap with those used in our previous studies (Miura et al. 2019).

### Statistical analysis

Since NAION is a rare disease, the number of cases that can be enrolled within the study period was set. Due to an exploratory study with a small number of subjects, no prior power calculation was performed.

Demographic patient characteristics included age and sex by median, range (minimum to maximum), and frequencies. Clinical characteristics are presented as mean and standard deviation.

The primary and secondary endpoints were analyzed in the full analysis set, defined as all patients who underwent at least one TdES session and had at least one efficacy assessment. The results are presented as mean and mean difference from the baseline, 95% confidence intervals (CIs).

## Results and discussion

**Fig. 2:**
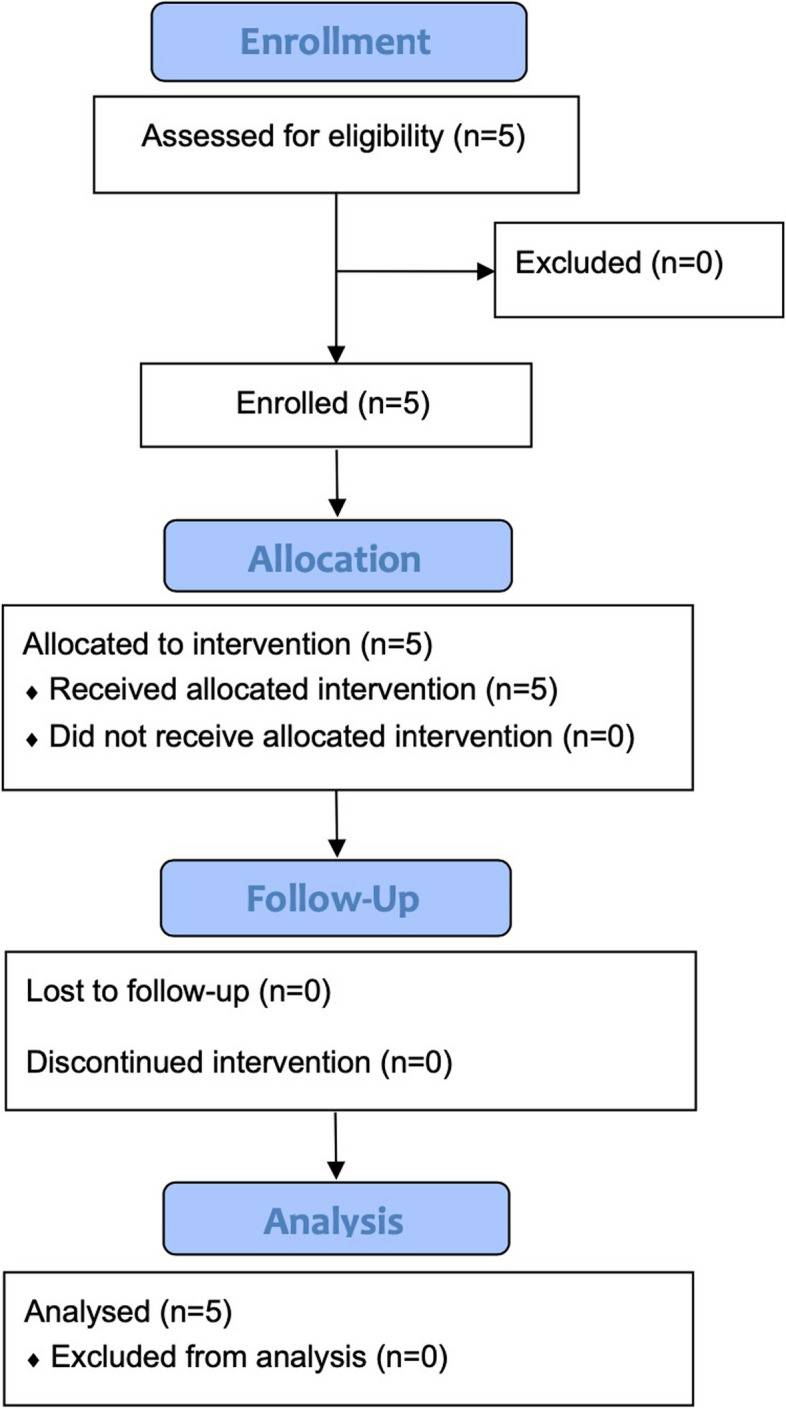
Study CONSORT flowchart

Five patients were screened, and no patient was excluded due to violation of the inclusion criteria or meeting the exclusion criteria. None of the cases were discontinued after enrollment. Finally, five eyes from five patients were included in the statistical analyses (Fig. 2).

The study was performed in accordance with the relevant guidelines and regulations. The demographic and clinical characteristics of the patients at baseline and 12 weeks are shown in Table 2.

**Table 2:** Demographic and clinical characteristics at baseline and 12 weeks

Parameters	Baseline	12 weeks
Number of patients / eyes	5 / 5	
Age (years)	72.6 (67 to 79)	
Male / Female	1 / 4	
logMAR VA	1.33 ± 0.39	1.26 ± 0.44
ETDRS VA (letters)	16.8 ± 16.75	16.4 ± 15.57
MD value of HFA 10–2 (dB)	-26.13 ± 6.89	-27.70 ± 6.34
Score of HFA Esterman test	37.75 ± 39.66	44.40 ± 36.40

Numerical values are presented as means ± standard deviation of the means

*Abbreviations*: *VA* Visual acuity, *MD* Mean deviation, *HFA* Humphrey field analyzer, *logMAR* Logarithm of the minimum angle of resolution, *ETDRS* Early Treatment of Diabetic Retinopathy Study

Only one patient underwent sub-Tenon triamcinolone acetonide injection at another hospital 4 days after onset (7 months before the introduction of TdES in this study). The other four patients had not been treated for NAION before inclusion in this study.

### Efficacy of this trial

**Fig. 3:**
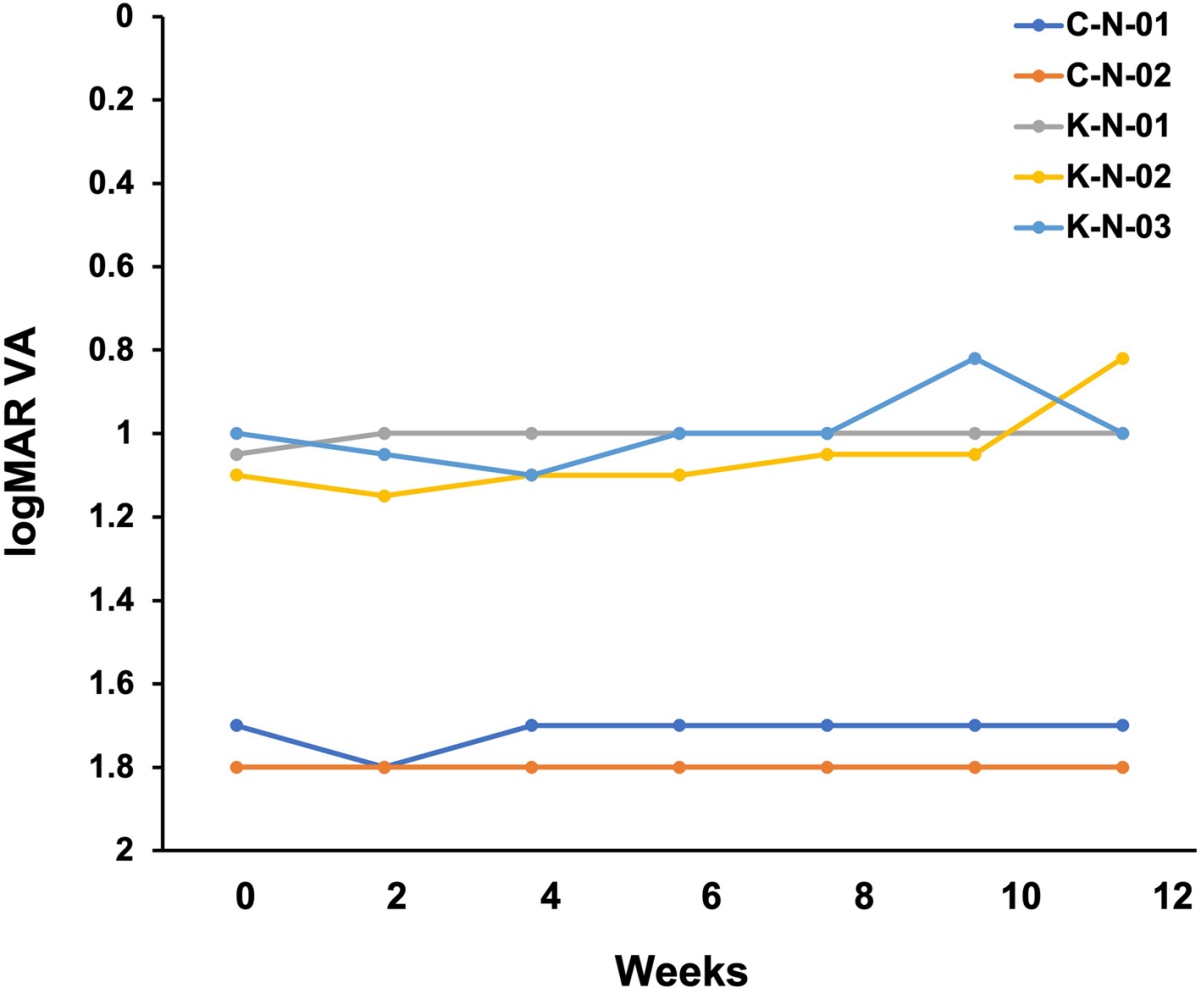
logMAR visual acuity for each case during the study period

logMAR visual acuity for each patient is shown in Fig. 3. In this study, logMAR visual acuity improved by ≥ 0.1 in two out of five eyes, and ETDRS visual acuity improved by ≥ 5 characters in one eye.

**Fig. 4:**
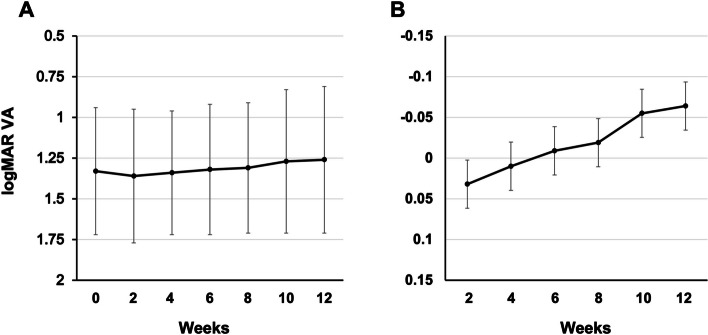
The mean and changes in logMAR visual acuity during the study period. **A** The mean logMAR visual acuity. Whiskers indicate standard deviation. **B** The mean changes in logMAR visual acuity. Whiskers indicate standard error

The mean ± standard deviation and changes in logMAR visual acuity from the baseline (0 weeks) throughout the study period (12 weeks) are shown in Fig. 4.

The mean logMAR visual acuity at baseline and at 2, 4, 6, 8, 10, and 12 weeks was 1.33 ± 0.39, 1.36 ± 0.41, 1.34 ± 0.38, 1.32 ± 0.40, 1.31 ± 0.40, 1.27 ± 0.44, and 1.26 ± 0.45, respectively (Fig. 4A).

In the mixed-effects model for comparison of logMAR visual acuity with that at week 0, the least mean square ± standard error was -0.032 ± 0.0296 (95% CI, -0.030–0.094) at 2 weeks, 0.010 ± 0.0296 (95% CI, -0.052–0.072) at 4 weeks, -0.009 ± 0.0296 (95% CI, -0.071–0.053) at 6 weeks, -0.019 ± 0.0296 (95% CI, -0.081–0.042) at 8 weeks, -0.055 ± 0.0296 (95% CI, -0.116–0.007) at 10 weeks, and -0.064 ± 0.0296 (95% CI, -0.126– -0.002) at 12 weeks (Fig. 4B).

Table 2 shows values of the logMAR visual acuity, ETDRS visual acuity, MDs of the HFA 10–2 and HFA Esterman scores at baseline and the final visit.

The mean ETDRS visual acuity was 16.8 ± 16.75 letters at baseline and 16.4 ± 3.91 letters at 12 weeks. The mean change ± standard deviation was -0.40 ± 3.91 (95% CI, -5.26–4.46). One patient (K-N-03) showed an improvement in the ETDRS visual acuity of ≥ 5 letters.

The mean MD of HFA 10–2 was -26.13 ± 6.89 dB at baseline and -27.70 ± 6.34 dB at 12 weeks. The mean change ± standard deviation was -1.58 ± 6.91 (95% CI, -10.15–7.00) dB.

The mean HFA Esterman score was 37.75 ± 39.66 at baseline and 44.40 ± 36.40 at 12 weeks. The mean change ± standard deviation was -1.75 ± 1.50 (95% CI, -0.64–4.14). The HFA Esterman score improved in four out of five eyes.

### Safety of this trial

Adverse events occurred in 1 of the 5 eyes (20%) during the study period. This single-eye adverse event was mild ‘eye strain’. As there was no recurrence after electrotherapy, a causal relationship with electrostimulation treatment was ruled out. There were no adverse events for which a causal relationship with the study device could not be ruled out, serious adverse events, or deaths during the study period. Similar to our previous results with TdES (Miura et al. 2019), abnormalities such as keratitis, dermatitis around the skin electrodes, inflammation of the anterior ocular segment, opacities of the optic media, abnormalities of the fundus and facial and trigeminal nerves, and nasal abnormalities were not observed. There were no significant differences in the intraocular pressure between the baseline and final visits.

To the best of our knowledge, this study is the first clinical trial to assess the efficacy and safety of TdES using a skin electrode in patients with NAION. Similar to our previous results (Miura et al. 2019), skin irritation caused by electrical stimulation from the skin electrodes was tolerable in all five cases.

As described in the Background section, functional activation of RGC by increasing the level of insulin-like growth factor -1 (Morimoto et al. 2005), ciliary neurotrophic factor, brain-derived neurotrophic factor (Ni et al. 2009) fibroblast growth factor-2 (Sato et al. 2008), secretion of glutamine synthetase (Wang et al. 2011), intracellular levels of adenosine monophosphate (Morimoto et al. 2002); upregulation of Bcl-2 expression and downregulation of Bax expression (Ni et al. 2009); inhibition of the NF-kB signaling pathway; and suppression of microglial activation (Fu et al. 2018); decreased expression of IL-6 and cyclooxygenase-2 accompanied by increased expression of IL-10 (Fu et al. 2018); inhibition of IL-1b (Zhou et al. 2012) and microglia-derived tumor necrosis factor-α (Yin et al. 2016) have been proposed as mechanisms for improvement of visual function by ocular electrical stimulation. Therefore, ocular electrical stimulation treatment is thought to be effective for NAION, because in patients with NAION, the optic nerve and RGC are affected due to impaired blood flow in the posterior ciliary artery.

However, reports of electrostimulation therapy for NAION are scarce. Osako et al. reported that TES preserved the decreasing scotopic threshold response amplitude and RGC in a rodent model of NAION (Osako et al. 2013). Fujikado et al. performed TES for patients with NAION and traumatic optic neuropathy and reported an improvement in visual acuity (Fujikado et al. 2006). They performed electrical stimulation using a biphasic pulsed current of 600–750 μA (duration, 10 ms; frequency, 20 Hz; 20 pulses; 30 min) using a corneal electrode. The duration from the onset to TES treatment was 4–24 months (median, 6 months). Their results showed that the BCVA at 3 months after treatment improved by ≥ 0.3 logMAR units in two eyes and was unchanged in one eye. The area of the peripheral visual field improved significantly in one eye, was unchanged in one eye, and worsened in one eye at 3 months after TES.

The electrical stimulation settings used were different from ours, and the small sample sizes of both studies precluded a simple comparison. However, in both studies, the effects of electrical stimulation differed among individuals, improvement was not dramatic, and peripheral vision improved in some cases.

Electrical stimulation therapy is thought to be effective when a patient’s RGC are alive but nonfunctional or when progenitor-cell plasticity is retained (Kurimoto et al. 2020). Phosphenes are visual perceptions induced by stimuli other than light. Electrically evoked phosphene thresholds have been used as indicators of electrical stimulation of RGC in the evaluation of implanted artificial retinas. Naycheva et al. investigated electrical phosphene thresholds in healthy participants and patients with retinal diseases and glaucoma and showed that the electrically evoked phosphene threshold at 20 Hz was the lowest in healthy participants (0.062 ± 0.038 mA) and highest in patients with retinal artery occlusion (0.988 ± 1.142 mA), followed by patients with Stargardt’s disease (0.102 ± 0.097 mA), primary open-angle glaucoma (0.127 ± 0.09 mA), NAION (0.244 ± 0.126 mA), and RP (0.371 ± 0.223 mA) (Naycheva et al. 2012). These results suggest that electrical stimulation therapy may be effective for NAION and RP.

Considering the mechanism of electrical stimulation, a stronger therapeutic effect is expected when electrical stimulation treatment is introduced early after onset, since more RGC survive. However, a natural-history survey of NAION reported the possibility of spontaneous recovery within 6 months of onset (Hayreh and Zimmerman 2008). Therefore, to exclude spontaneous improvement, we set the criteria for inclusion in this clinical trial as cases with onset ≥ 6 months earlier. Thus, different results might have been obtained if electrical stimulation was administered earlier after onset. Further discussion and investigation are necessary regarding the intervention and effects of electrical stimulation therapy in the early post-onset period.

In this study, the mean ETDES visual acuity was 16.80 at baseline and 16.40 at 12 weeks. Regarding ETDRS vision, two, one, one, and one patients had baseline and 12-week scores of 0 and 0 letters, 39 and 33 letters, 23 and 28 letters, and 22 and 21 letters, respectively. In the patient in whom ETDRS vision improved from 23 to 28 characters, logMAR visual acuity also improved from 1.10 to 0.82 during the study period. However, in the patient in whom ETDRS vision reduced from 39 to 33 characters, logMAR visual acuity, MD of HFA, and other ophthalmic findings did not worsen during the study period. In addition to the above-mentioned visual acuity charts and visual-acuity conversion problems, poor reproducibility due to central visual acuity loss, unfamiliarity with visual-acuity measurement using the ETDRS visual-acuity chart, and differences between single-optotype visual acuity and multiple-optotype visual acuity are also considered to be causes of the difference in results depending on the measurement method. However, the exact cause of this remains unknown. It is necessary to establish appropriate evaluation methods for low-vision cases, such as three-dimensional visualization and volumetric sensitivity in microperimetry, which have been studied in recent years (Josan et al. 2021), and interpret the dissociation of visual acuity and visual field results.

For safety evaluation, electrostimulation was performed according to the protocol for all five patients. There were no cases of withdrawal due to stimulation or pain due to electrical stimulation. No adverse events related to the treatment were observed during the study period. Thus, we can conclude that TdES is safe under the implemented stimulation protocol.

This study had some limitations, such as the small number of cases, lack of a control group, and short treatment and observation periods. In addition, as mentioned above, the effect on NAION early after onset, which is expected to be greater, was not verified, and the phosphene threshold, an index of residual visual function, was not measured.

## Conclusion

In conclusion, this exploratory study of five patients with NAION may have suggested improvement in the mean change in logMAR visual acuity. The logMAR visual acuity may have been slightly improved in two patients and HFA Esterman score may have improved in four eyes. No serious adverse events occurred, and the treatment was tolerated in this study and our previous study in patients with RP (Miura et al. 2019).

Although it is difficult to discuss the efficacy in detail based only on the data obtained from this study, since NAION has no established treatment method, further sham-controlled study in larger cohort into the effects of TdES treatment in NAION patients may be beneficial.

### Supplementary Information

**Additional file 1.**

**Additional file 2.**

## Data Availability

The datasets generated and/or analyzed during this study are available from the corresponding author on reasonable request.

## Abbreviations

TdES: Transdermal electrical stimulation
NAION: Non-Arteritic Anterior Ischemic Optic Neuropathy
ETDRS: Early Treatment of Diabetic Retinopathy Study
HFA: Humphrey field analyzer
RP: Retinitis pigmentosa
RGC: Retinal ganglion cell
MD: Mean deviation
ESR: Erythrocyte sedimentation rate
BCVA: Best corrected visual acuity
CIs: Confidence intervals
