# Supplementary material for: Efficacy and safety of transdermal electrical stimulation in patients with nonarteritic anterior ischemic optic neuropathy
Source: Bioelectron Med. 2023 Oct 25;9:22. doi: 10.1186/s42234-023-00125-2 (PMC10598888; doi:10.1186/s42234-023-00125-2)
Supplement: Supplementary file 1 — Additional file 1. [file 42234_2023_125_MOESM1_ESM.docx]

**Clinical trial protocol**

**A study on the safety and efficacy of transcutaneous electrical stimulation for non-arteritic ischemic optic neuropathy**

**(Phase II / III study)**

| **Version** | **draft 2.0** |
| --- | --- |
| **Date** | **November 30th. 2018** |

**Rivision history**

| Date | Version |
| --- | --- |
| October 11^th^ 2018 | draft 1.0 |
| October 29^th^ 2018 | draft 1.1 |
| November 12^th^ 2018 | draft 1.2 |
| November 30^th^ 2018 | draft 2.0 |
|  |  |

UMIN ID：UMIN000036219

**Precautions regarding confidential information**

This clinical trial implementation plan is confidential information and will be provided to the investigators, investigators, sponsors, conducting medical institutions, clinical trial review committees, and efficacy safety evaluation committees who participate in this clinical trial.

Therefore, except when explaining to the subject, it cannot be disclosed to any third party or used for purposes other than this clinical trial without the written consent of the investigator and Mayo Co., Ltd., the provider of the investigational device.

In addition, if you want to publish part or all of the results of this clinical trial to outside academic societies, magazines, etc., the consent of the investigator and Mayo Co., Ltd. is required, so please contact the clinical trial coordinating doctor in advance.

＜Abbreviations＞

| AC | alternating current |
| --- | --- |
| Ag | argentum |
| ALT | alanine aminotransferase |
| ALP | alkaline phosphatase |
| AST | aspartate aminotransferase |
| BDNF | brain-derived neurotrophic factor |
| BUN | blood urea nitrogen |
| CNTF | ciliary neurotrophic factor |
| COX-2 | cyclooxygenase-2 |
| CRP | C-reactive protein |
| CRAO | central retinal artery occlusion |
| dB | decibel |
| DC | direct current |
| ERG | electroretinogram |
| ESR | erythrocyte sedimentation rate |
| ETDRS | early treatment diabetic retinopathy study |
| FAS | full analysis set |
| FGF1 | fibroblast growth factor |
| GOT | glutamic oxaloacetic transaminase |
| GP | goldmann perimetry |
| HFA | humphrey field analyzer |
| GPT | glutamic-pyruvic transaminase |
| HbA1c | hemoglobin A1c |
| Hz | hertz |
| IGF-1 | insulin-like growth factors |
| IL-1b | interleukin-1b |
| IL-6 | interleukin-6 |
| IL-10 | interleukin-10 |
| IS/OS | photoreceptor inner/outersegment junction |
| LD50 | lethal dose 50 |
| mA | milliampere |
| MD | mean deviation |
| mmHg | millimeter Hg |
| msec | milli second |
| NAION | non-arteritic ischemic optic neuropathy |
| NGSP | national glycohemoglobin standardization program |
| NF-κB | nuclear factor kappa-light-chain-enhancer of activated B cells |
| PPS | per protocol set |
| RP | retinitis pigmentosa |
| TdES | transdermal electrical stimulation |
| TES | transcorneal electrical stimulation |
| TNF-α | tumor necrosis factor-α |

# 0. Overview

| Title | A study on the safety and efficacy of transcutaneous electrical stimulation for non-arteritic ischemic optic neuropathy (Phase II / III study) |
| --- | --- |
| Purpose | To confirm the safety and efficacy of transcutaneous electrical stimulation using skin electrodes for patients with non-arteritic ischemic optic neuropathy for visual function after treatment. |
| Design | prospective, non-randomized, open-label, uncontrolled multicenter trial |
| Phase | Ⅱ/Ⅲ |
| Device | 1. Overview   This device is that a skin electrode for electrical stimulation is attached to the center of the forehead and the skin on the lower eyelid ear side of the target eye to give constant electrical stimulation under various conditions to treat retinal and optic nerve diseases.  Constitution  　It consists of an "electrical stimulator", a "skin electrode" and an "electrode connection cable" that connects them.   1. Specification 2. Electrical stimulation device  - Current 0～3 mA   ・Time 0～10 msec×2 biphasic  ・Inverval 50 msec   1. Skin electrode　（Sekisui Kasei Co.Ltd.）   ・Size　　　　19×38 mm  ・Element　　　Ag/Agcl  ・Base material　　　　　Non-woven fabric  ・Gel 　　　　Conductive adhesive gel   1. Capacitance   AC adapter rating (input)　AC 100 V　50/60 Hz　30 VA  AC adapter rating (output)　DC 5 V　4 A   1. Weight：700 g、Size： 225×165×40 mm |
| Inclusion Criteria | 1. Clinically diagnosed with NAION and age ≥20 years and ≤80 years  2. NAION patients who were determined to have fixed symptoms more than 6 months after the onset  3. Decimal visual acuity from hand motion to 0.7  4. Patients who provided signed consent with sufficient understanding after receiving an explanation of the responsibilities of participating in this trial  5. Regular hospital visits every 2 weeks for 12 weeks |
| Exclusion Criteria | 1. Any of the following on a screening blood sampling test:  ・ ESR: more than 20 mm/h  ・ CRP: more than 10 mg/l  2. Patients with ocular disease other than ischemic optic neuropathy confirmed by contrast-enhanced MRI at the time of definitive diagnosis of non-arteritic ischemic optic neuropathy.  3. History of allergy to mydriatic agents and eye surface anesthetics  4. Presence of vitreous macular traction syndrome, macular edema, epiretinal membrane, myopia with posterior staphyloma, diabetic retinopathy, conjunctival inflammation, ocular infection, severe dry eye, grade 3 or higher Emery-Little grade cataract, and posterior capsule opacification  5. History or complications of a malignant tumor. However, patients with a history of malignant tumor that has not relapsed for more than 5 years were not excluded  6. Diagnosis of dementia or mental disorder with ongoing treatment  7. Diabetes mellitus (HbA1c [NGSP]> 10.0%)  8. Hypertension (systolic ≥ 180 mmHg and/or diastolic ≥ 110 mmHg) that is difficult to control even with oral treatment  9. Any of the following on a screening blood sampling test:  ・ Aspartate aminotransferase and alanine aminotransferase: more than three times the upper limit of the facility standard value  ・ Serum creatinine: more than 1.5 times the upper limit of the facility standard value  10. Ongoing treatment with ethambutol hydrochloride and/or amiodarone hydrochloride  11. Ongoing pregnancy, breastfeeding, or possible or planned pregnancy during the trial period  12. Participation in other clinical trials  13. Under investigational responsibility (shared) judged by doctors to be inappropriate for participation in this trial |
| Endpoints | ＜Primary endpoint＞  The logarithm of the minimum angle of resolution (logMAR) visual acuity  ＜Secondary endpoint＞  1. Changes in logMAR visual acuity  2. Changes in Early Treatment of Diabetic Retinopathy Study (ETDRS) visual acuity  3. Changes in mean deviation value of HFA 10-2  4. Changes in ccore of HFA monocular Esterman test scores  ＜Safety endpoint＞  The incidence of adverse events (AEs; type, frequency, and severity) |
| Methods | This clinical trial consists of a pre-observation period and a clinical trial period.  The clinical trial period will be 12 weeks, and transdermal electrical stimulation treatment using skin electrodes will be performed 6 times every 2 weeks. The stimulation conditions during this treatment are as follows.  ＜Stimulation conditions＞   - Current strength：1.0 mA - Pulse duration：10 ms/phase - Frequency：20 Hz - Time：30minutes |
| Discontinuance criteria | 1) When the subject offers to decline participation in the clinical trial or withdraws consent.  2) When the subject is found to be ineligible for the subject after enrollment in the clinical trial.  3) When it is difficult to continue the clinical trial due to exacerbation of complications.  4) When it is difficult to continue the clinical trial due to adverse events.  5) If pregnancy is found.  6) If the entire clinical trial is discontinued.  7) When other serious violations of the clinical trial protocol are found.  8) When the necessity of discontinuation is recognized at the discretion of the investigator or the investigator. |
| Target number | 5 eyes of 5 patients |
| Trial period | Implementation period： 15 months （April 1. 2019～June 30. 2020）  Registration period： 12 months 　（April 1. 2019～March 31. 2020） |
| Facilities number | 3 |
| Ethics | Informed consent was obtained from all the patients for the electrical stimulation and other examinations. Written informed consent was obtained from all patients before enrollment. The study procedures conformed to the tenets of the Declaration of Helsinki. |
| The Institutional Review Boards | Prior to conducting this clinical trial, the Institutional Review Boards of the conducting medical institution will review the ethical, scientific and medical validity of this clinical trial. This clinical trial will be conducted after obtaining the approval of the Institutional Review Boards.  The Institutional Review Boards will continuously review whether the study is being conducted properly at least once a year. |

# Backgound

In 1955, Brindley reported that applying an electric pulses to the eye produced a light sensation called a phosphene^1^. Then, Potts et al. reported that electrically-evoked potential changes could be recorded from the cranial surface at the same time as the phosphenes by electrical stimulation of the eye^2^. Thus, applications of electrical pulses to the eye could be used to investigate the origin of phosphenes and electric evoked responses (EERs) evoked by electrical stimulation. In the 1990’s, Galli-Resta et al reported that spontaneous afferent electrical activity regulated the targeted cell death in the developing rat visual system^3^. The results also showed that short periods of low frequency electrical stimulation accelerated axonal regeneration of peripheral neurons by Al-Majed et al^4^. Morimoto et al reported that the survival rate of rat retinal ganglion cells (RGCs) after transection of the optic nerve was significantly higher in rats whose optic nerve was electrically stimulated than that of the untreated group^5^. Morimoto et al. also found that electrical stimulation had a neuroprotective effect on RGCs, and they developed a transcorneal electrical stimulation device (TES) that could be used in humans^6^. They used a contact lens type of electrode that is used to record electroretinograms (ERGs), and the electrical pulses were obtained from an electrical stimulation device and delivered through the electrodes embedded in the contact lens. They studied the effects of TES on its neuroprotective activity on optic nerve diseases. Thereafter, it was reported that TES can protect the retinal photoreceptors in animal models of RP of rats and rabbits^7, 8^. Also, in clinical studies on central retinal artery occlusion(CRAO) patients, TES improved the visual acuity, visual fields, and ERGs after TES has also been reported^9^.

Previous TES studies used the contact lens type or TDL fiber type corneal electrodes, whereas the electrical stimulation device used in this study was a patch containing the electrode that is applied to the skin, and electric stimulation is delivered through the skin so that it is less invasion and can be done more easily. This transdermal electrical stimulation (TdES) was performed using prototype equipment developed jointly with the Mayo Co., Ltd.

Thus, the purpose of this clinical trial was to verify the safety and efficacy of TdES using skin electrodes for patients with CRAO and evaluate the visual functions before and after the TdES.

The mechanisms proposed for the improvement of visual functions include an increase in the level of IGF-1^6^, ciliary neurotrophic factor (CNTF) and brain-derived neurotrophic factor (BDNF)^10^, up regulation of Bcl-2 expression and down regulation of Bax expression^10^, increase in the chorioretinal blood flow^11^, inhibition of the NF-kB signaling pathway, and suppression of microglia activation^12^, up regulation of 25 proteins included cellular signaling proteins, proteins associated with neuronal transmission, metabolic proteins, immunological factors, and structural proteins^13^.

It has been reported that TES using corneal electrodes improved the visual function of eyes with traumatic and ischemic optic neuropathy^14^, retinal artery occlusion^16^.

The pathology of NAION is damage to the inner retina, including the optic nerve and ganglion cells, due to occlusion of the posterior ciliary artery, a branch of the ophthalmic artery. Stimulation therapy is considered to be a therapeutic method that is expected to improve visual function in NAION as well.

Small portable devices have already been commercialized overseas, and mainly in Europe, it is an electrical stimulation device that uses skin electrodes that cover the eyelids and skin electrodes on the back of the hand, targeting retinal diseases such as RP and age-related macular degeneration. Although "ScyFix®︎" and "OkuStim®︎", an electrical stimulator using contact lens-type corneal electrodes, are used clinically, they are not commercialized in Japan.

In addition, TES's "OkuStim®︎" is easy to operate because electrodes are placed on the cornea to perform electrical stimulation. pointed out. In contrast, the electrical stimulation treatment used in this study is considered to be less invasive and easier to perform electrical stimulation than corneal electrodes by placing electrodes on the skin of both cheeks and forehead.

We performed transdermal electrical stimulation (TdES) using skin electrodes placed on both cheeks and forehead 6 times at 2-week intervals in 20 eyes of 10 patients with RP. In an exploratory study (phase II study) to investigate the safety and efficacy of transcutaneous electrical stimulation for degeneration, no serious adverse events or problems occurred.

Adverse events occurred in 4 subjects (cold in 4 subjects, diarrhea in 1 subject, fever in 1 subject), but no causal relationship to the study device was observed.

On the other hand, in the study of efficacy, ETDRS visual acuity showed statistically significant improvement at 6 weeks, 10 weeks and 12 weeks compared to 0 weeks, and an average improvement of about 4 letters was observed at 12 weeks. LogMAR visual acuity also showed a statistically significant improvement after 8 weeks compared to 0 weeks. The Mean Deviation value (MD) in static visual field testing showed an improvement trend, and a statistically significant difference was observed at 12 weeks from 0 weeks.

Since safety was confirmed in the above clinical trial results and an improvement effect on visual function was observed, it is conceivable that NAION may also improve visual acuity and visual field due to the neuroprotective effect of TdES.

TdES is less invasive and may be a treatment with less economic burden, and it is considered clinically useful, so we decided to consider the development of this treatment method using skin electrodes. At present, there is no established treatment method, and once it develops, visual field impairment due to severe optic nerve damage occurs, and the quality of life is significantly reduced. Therefore, we planned this trial.

## **Standard treatment for central retinal artery occlusion**

Regarding the treatment of NAION, there is currently no clear effect on visual acuity improvement, and the establishment of a treatment method is an important issue.

**Overview of clinical trial equipment**

## In addition to controlling the intensity (current amount) and time (treatment time) of electrical stimulation, the treatment eye (right eye, left eye, binocular) is selected. The main body displays the magnitude of the current energized by electrical stimulation, the energization time, and the like. The skin electrode contacts the skin on the surface with the gel-like adhesive substance, and the cable connecting the skin electrode and the main body is connected to the protrusion on the opposite surface. The electrodes should be attached to the lower eyelid ear side of the treated eye and the center of the forehead.

| The electrodes on the gel-like pad are similar to the disposable electrodes used in home low frequency therapy devices. The size is about 19 x 38 mm, and the surface with the gel-like adhesive substance comes into contact with the skin, and the cable connecting the electrode and the device is connected to the protrusion on the opposite surface, and electrical stimulation is applied via the skin. Will be done. The electrodes should be attached to the lower eyelid ear side of the treated eye and the center of the forehead (right figure). | 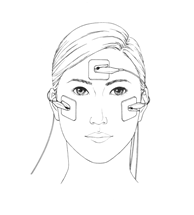 |
| --- | --- |

###

### **Non-clinical results**

Since the electrode pad used for the device comes into contact with healthy skin, it is required to guarantee the biological safety. According to the biological test data of Sekisui Kasei Kogyo Co., Ltd., the manufacturer of electrode pads, it is said that there are no adverse events related to biosafety in biological hydrogels (Technogel^®^). It is used for electrodes for brain waves, biosensors, electrodes for electrotherapy devices, etc. The outline of the test data is described below.

【Biological test data（SRA240/80-09S）】

1. Biological tests were conducted based on the following standards.

| Test | Standards and methods |
| --- | --- |
| Primary skin irritation test | ISO 10993-10:2010 - Biological evaluation of medical devices Part 10: Test of irritant and delayed hypersensitivity (sectiuon 6.3) |
| Sensitization test | ISO 10993-10:2010 - Biological evaluation of medical devices Part 10: est of irritant and delayed hypersensitivity (sectiuon 7.2)  ISO 10993-12:2012 - Biological evaluation of medical devices Part 12: Sample preparation and target substances |
| Cytotoxicity test | ISO 10993-5:2009 - Biological evaluation of medical devices Part 5: Cytotoxicity test: in vitro |
| Kawai method.  "24-hour openness coating test" | A sample is applied to the medial part of the upper arm of 20 subjects for 24 hours, and the application position is observed. If there is no abnormality with the naked eye, a replica specimen is prepared and the entire visual field is observed under a microscope to determine the presence or absence of irritation and the intensity. |

1. Results（SRA240/80-09S）

| Test Method | Result |
| --- | --- |
| Cytotoxicity test non-contact (agar) method | No cytotoxicity |
| Local Limba node assay using mice | Negative |
| Primary skin irritation test using rabbits | P.I.I=0, No irritation |
| Kawai method. "24-hour openness coating test" | 3B-0C-0D、Extremely weak to humans |

**Animal model studies**

| Reference | Subject | Electrical stimulation pathway | Condition | Result |
| --- | --- | --- | --- | --- |
| Sato T et al  (2008)^1^ | 1 Subcultured Muller cells (12-14 day old Long-Evans rats) | In vitro | 1ms, 20Hz, 0, 1, 5. 10 mA, 30 minutes | Muller intracellular IGF-1 mRNA increase  Increased intracellular influx of Ca2 + from L-type Ca2 + channels |
| Sato Tet al  (2008) ^6^ | 1 Subcultured Muller cells (12-14 day old Long-Evans rats) | In vitro | 1ms, 20Hz, 0, 1, 5. 10 mA, 30 minutes | Increased Muller Cell Brain-Derived Neurotrophic Factor (BDNF) mRNA  Muller Cellular BDNF Increase |
| Morimoto T et al (2002) ^2^ | Optic nerve transaction Slc:Wistar Rat | Optic nerve amputation end | 20Hz, 20, 30, 50, 70 μA, 2hours | Neuroprotective effect network on retinal ganglion cells (RGC) (RGC survival rate increases with current intensity) |
| Morimoto T et al (2005) ^5^ | Optic nerve transaction Slc:Wistar Rat | Transcorneal | 20Hz, 100μA, 1hours | Increased survival of retinal ganglion cells (RGC),  Increased IGF-1 production in Muller cells in the retina |
| Willmann G et al (2011)^19^ | Brown Norway Rat | Transcorneal | 20Hz, 100μA, 1hours | Downregulation of Bax and specification necrosis factor (TNF) family (Tnfrsf11b, Tnrsf12a, Tnrsf13b, Tnrsf13) associated with retinal cell apotosis |

**Clinical Studies**

| Schatz A et al^18^ | TES once a week for 30 minutes 6 times for 24 RP cases, no problem with safety and tolerability, efficacy: visual field and dark adaptation b wave significantly improved. |
| --- | --- |
| Schatz A et al^15^ | 52 patients with RP, once a week for 30 minutes for 1 year, safety results: transient dry eye symptoms (31 of 52), no other clinically significant adverse events. Effectiveness result: Light-adaptive b-wave is significantly improved, and dark-adaptive b-wave amplitude is improving. |
| Bittner AK et al^19^ | 3 patients with retinitis pigmentosa who received TES 3 to 6 times over 2.4 to 3 years 1, Safety: No treatment-related adverse events, Efficacy: Improvement of central vision function repeated about 4 to 7 weeks after TES It was admitted, then returned to baseline, and there was no significant decline in visual function beyond baseline. |
| Inomata K et al^20^ | CRAO2 eyes, BRAO1 eyes, VA and mfERG improved in 2 cases, and VF improved in all 3 cases. |
| Oono S et al^21^ | BRAO long-term 2 eyes, initial diagnosis 3 eyes, efficacy; significantly improved in VA2 eyes, no change 1 eye, 1 eye, P1 latency significantly shortened. |
| Fujikado T et al^14^ | Improvement of visual acuity was observed in 3 cases of NAION, 5 cases of TON (traumatic optic neuropathy), 2 cases of NAION and 4 cases of TON. |

**Expected adverse events and malfunctions, etc.**

Although it is presumed that adverse events are unlikely to occur based on the amount of current used, dermatitis and corneal damage that match the electrode mounting site may occur. In addition, it is expected that a feeling of irritation will be felt on the skin near the electrode mounting site due to electrical stimulation. In addition to the defect information of medical devices, the defect information due to improper usage (immature procedure) is also included.

# **Subject consent**

## The investigator should prepare consent documents and other explanatory documents used to obtain consent from the subject to participate in the study in the simplest possible language. In addition, if we find it necessary to revise the consent document and other explanatory documents, we will revise them.

The investigator submits the prepared or revised consent document and other explanatory documents to the clinical trial review committee for approval.

## **When and how to get consent**

## The investigator or the investigator will hand the consent document and other explanatory documents approved by the clinical trial review committee to the subject, and give a sufficient explanation of the contents shown in "4.3 Explanations to the subject". If necessary, the study collaborators will also provide supplementary explanations to the subjects. After confirming that the subject has a good understanding of the content of the clinical trial, obtain written and voluntary consent before conducting the pre-observation period (screening) test.

## 1) Correspondence to the subject at the time of explanation

## The investigator or investigator should give the subject sufficient time to ask questions and decide whether or not to participate in the trial before obtaining consent, and the subject's questions should be answered by the subject. Answer to be satisfied.

## 2) How to fill out the consent form and issue an explanatory document

## Upon consent of the subject, the investigator or investigator who gave the explanation shall sign or sign the name and enter the date of explanation. The subject signs or signs the consent form and writes the date of consent. If the study collaborator gives a supplementary explanation, the study collaborator will also sign or sign the name and enter the date of the explanation. After obtaining consent, a copy of the explanatory document and consent form will be delivered to the subject.

## 3) When the explanatory document is revised

Whenever the investigator or the investigator revise the consent document and other explanatory documents due to the acquisition of new information that may be related to the consent of the subject, the revised consent document and other documents will be given to the subject. Explain again using explanatory documents, and obtain written voluntary reconfirmation from the subjects regarding the continuation of participation in the clinical trial. If new important information that may affect the consent of the subject is obtained, immediately provide the information to the subject, record it in writing, and whether the subject will continue to participate in the clinical trial.

## **Informed consent to the subject**

## The explanatory document prepared by the investigator should include the following items:

## 1) The clinical trial involves research

## 2) Purpose of the clinical trial

## 3) Name, job title and contact information of the investigator

## 4) Clinical trial method

## 5) Expected clinical benefits and risks or inconveniences

## 6) Presence or absence of other treatments for the subject and the expected significant benefits and risks associated with those treatments.

## 7) Scheduled period for participating in the subject's clinical trial

## 8) Participation in the clinical trial is at the discretion of the subject, and the subject may refuse or withdraw from the clinical trial at any time. In addition, the subject will not be treated unfavorably due to refusal or withdrawal, and will not lose the benefits that should be received if he / she does not participate in the clinical trial.

## 9) Monitors, auditors, clinical trial review committees and regulatory agencies have access to medical sources. At that time, the subject's confidentiality must be preserved.

## 10) Subject confidentiality should be preserved even if the results of the clinical trial are published.

## 11) Compensation and treatment that subjects can receive in the event of clinical trial-related health hazards

## 12) Promptly inform the subject of any information that may affect the subject's will regarding continued participation in the clinical trial.

## 13) Conditions or reasons for discontinuing participation in clinical trials

## 14) Costs borne by the subject regarding the clinical trial

## 15) If money, etc. is paid to the subject regarding the clinical trial, the details (arrangement for calculating the payment amount, etc.)

## 16) A medical institution consultation desk that should be inquired or contacted if the subject wants more information about the clinical trial and the subject's rights or if there is a health hazard related to the clinical trial.

## 17) Matters to be observed by the subject

## 18) Types of clinical trial review committees that conduct investigations and deliberations on the suitability of the clinical trials, matters to be investigated and deliberated by each clinical trial review committee, and other matters related to the clinical trial review committees related to the clinical trials.

## 19) Intellectual property

20) Conflict of interest

Facility registration and case registration will be conducted by the central registration system in the Data Management Office, Clinical Trials Department, Chiba University Hospital.

1) The investigator will fax a copy of the approval notice of the clinical trial review committee and the facility registration request form to the case registration center after approval is obtained by the clinical trial review committee of the facility.

2) The Case Registration Center will register the facility and send a notification of completion of facility registration to the investigator.

1) The chief investigator or co-investigator obtains written consent and, as a result of the screening test, confirms that the subject meets the selection criteria and does not violate the exclusion criteria. The chief investigator, the co-investigator or collaborator will register the subject that the investigator or the investigator judges to be "qualified". Case registration is done on the website.

2) The chief investigator, co-investigator or collaborator will access the specified URL and enter the information required for case registration on the website. The chief investigator or co-investigator confirms the eligibility determination on the screen, and if it is determined to be eligibility, the protocol treatment is started. Once registered, the registration of the subject will not be cancelled. In the case of duplicate registration, the initial registration information (registration number) will be adopted in any case. If incorrect registration or duplicate registration is found, immediately contact the Data Management Office, Clinical Trials Department, Chiba University Hospital.

* The chief investigator or co-investigator must not perform protocol treatment until the subject is registered.

### **Facility registration / case registration destination**

Data Management Office, Clinical Trials Department, Chiba University Hospital

TEL：043-222-1206　　　　FAX：043-222-1207

Subjects who are not enrolled for any reason, such as ineligibility, are not included in the enrolled cases of the study. The investigator will explain to the subject that enrollment in this study is not possible.

# **Clinical Trial Equipment**

It consists of an electrical stimulator, a skin electrode (Yerrode®, Sekisui Kasei Co., Ltd.) and an electrode connection cable connecting them.

| 2. Electrode pad | 3. Electrode lead wire |
| --- | --- |
| 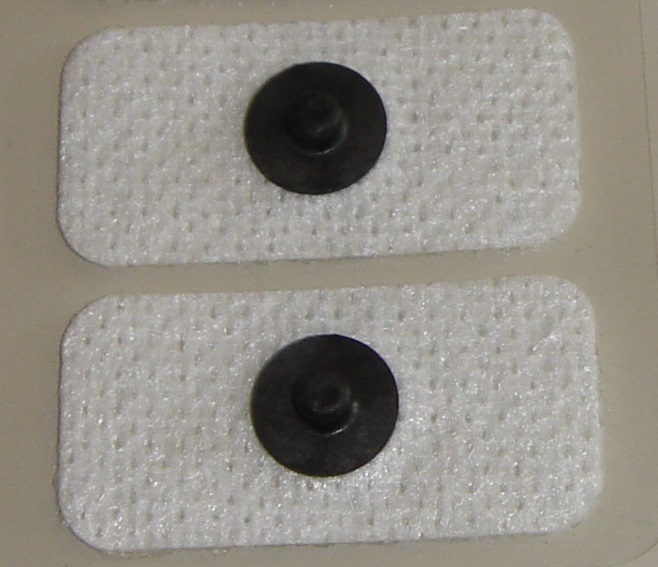 | 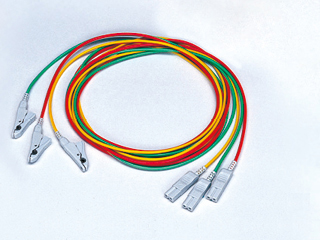 |

## The device is equipped with an emergency stop button.

The device is powered by an AC adapter. The AC adapter produces a DC 5 V power supply (DC 5 V) from a commercial AC power supply (100 VAC, 50/60 Hz). The AC adapter uses a switching method to generate a predetermined direct current from alternating current. In the switching method, direct current is generated through the following process.

1) 100 VAC is rectified to the positive side by a diode bridge rectifier and smoothed by a capacitor.

2) The current that is switched on / off at high speed on the primary side by a high frequency transformer is output to the secondary side.

3) A DC power supply is output after being smoothed through a rectifying diode.

The switching method has a complicated circuit configuration, but is composed of high withstand voltage components. The switching method is superior in terms of performance to the withstand voltage test required in the electrical safety test, and the AC adapter used in this device is also based on the switching method.

In the CPU circuit, the input from the presence or absence of the button switch for making 6 settings and whether or not the emergency stop button is pressed, and the input with the dial rotated clockwise / counterclockwise are written to the CPU. The program is executed by the installed firmware. The program execution status and the status of button switches and dials are displayed on a 16-character x 2-line liquid crystal display. The CPU is controlled by a 20 MHz crystal oscillator with an accurate clock.

The amount of current to be energized is accurately controlled by a constant current circuit and an 8-bit (256 steps) digital potentiometer (digital variable resistor) with a PNP transistor and an NPN transistor.

For positive and negative polar stimuli, On / Off information is input to the photocoupler by a logic circuit and output to three sockets that are optically isolated and have electrodes connected to the patient.

As for the power supply for electrical stimulation, DC 5 V is turned on / off at high speed and input to the transformer in a pulse shape, and the pulse waveform output by the transformer output is smoothed to generate DC 100 V.

Mayo Inazawa Research Institute Co., Ltd. is certified as a business establishment that complies with the QMS ministry ordinance set by the authorities, and is licensed as a medical device registered manufacturing facility. In addition, Cosmos Corporation (certification body number: AG), which is a certification body, has certified conformity to all certified medical devices by QMS periodic survey. Risk management is implemented for the investigational equipment in accordance with ISO14971, and the quality of the investigational equipment is guaranteed.

The investigational device is designed to comply with the following JIS standards for medical devices related to electrical safety.

| Number | Title |
| --- | --- |
| JIS T 0601-1 | Medical Electrical Equipment-Part 1: General Requirements for Basic Safety and Performance |
| JIS T 0601-1-2 | Medical Electrical Equipment-Part 1-2: General Safety Requirements-Electromagnetic Compatibility-Requirements and Testing |
| JIS T 0601-2-10 | Medical Electrical Equipment-Part 2-10: Individual Requirements for Neural and Muscle Stimulator Safety |

The withstand voltage test, leakage current test, low resistance test, and power consumption, which are the basic electrical safety tests, meet the requirements of the standard and are considered to ensure patient safety. The safety test will be conducted on mass-produced machines. At the time of this clinical trial, the attached AC adapter conforming to JIS T0601-1 and JIS T0601-1-2 was used, and it was not affected or affected by electromagnetic influences from other electronic devices. It will be implemented after taking safety measures such as ensuring the space distance.

Although it is presumed that adverse events are unlikely to occur based on the amount of current used, dermatitis and corneal damage that match the electrode mounting site may occur. In addition, it is expected that a feeling of irritation will be felt on the skin near the electrode mounting site due to electrical stimulation.

The accuracy required by JIS standard T0601-2-10 for medical devices is shown below.

Current value: 0 to ± 2,000 μA (accuracy rated output 0 to ± 30%)

Stimulation frequency: 20 Hz (accuracy ± 1%)

Output waveform: Both positive and negative phases

Pulse width: 5 to 10 msec for each phase (accuracy ± 1%)

**Manufacturing**

The scope of the quality control system for the item is the area surrounded by the thick line.

Materials, parts, components

The range surrounded by the dotted line is the process range of the following manufacturing plants.

Factory name:

Mayo Inazawa Research Institute Co., Ltd.

Factory location:

2-25-22 Takamido, Inazawa City, Aichi Prefecture

Manufacturing license number: 23BZ005022

Manufacturing license classification: General

Acceptance inspection of materials, parts and components

Manufacturing, processing and in-process inspection

Product inspection

Packaging/ labeling

Strage and shipping decision

Shipping

Packaging and labeling (Written in Japanese)

<Clinical trial device label (sample)>
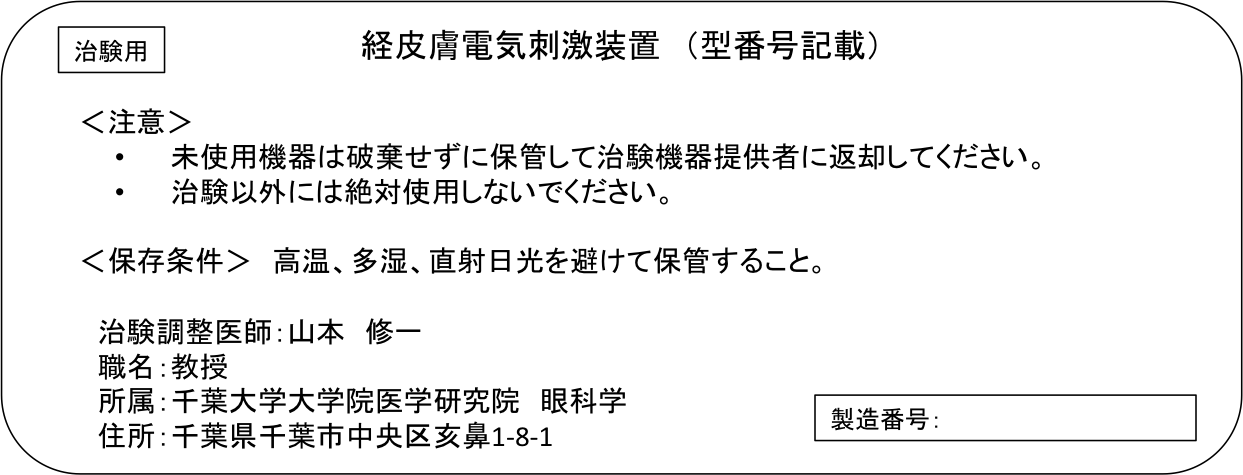


The label should include the title of the clinical trial coordinating physician and the serial number of the transcutaneous electrical stimulator.

**Management method**

1) The clinical trial coordinating doctor will deliver the clinical trial equipment to the investigator of the conducting medical institution immediately after the start of the clinical trial.

2) The clinical trial equipment manager of the clinical trial institution properly manages the clinical trial equipment at room temperature according to the procedure manual provided by the investigator through the director of the clinical trial institution.

3) The investigator prepares a document explaining the storage conditions for supplying the investigational device, the expiration date, etc. Will be delivered to.

4) The investigator and the investigator confirm the device number to be treated, and if there is a problem with the treatment device, including during non-treatment, report the device number and the status of the problem, and replace or repair it. We will respond promptly if necessary.

**Implementation schedule and procedure**

The schedule for observation, inspection, and evaluation is shown in the table below. The investigator or the investigator will carry out observations and examinations according to the schedule. Items that can be carried out by the investigator, such as subject background surveys and clinical tests, may be carried out by the investigator under the control of the investigator. (See the schedule below)

|  | **Screening** | **0 week** | **2 weeks** | **4 weeks** | **6 weeks** | **8 weeks** | **10 weeks** | **12 weeks** | **Drop out** |
| --- | --- | --- | --- | --- | --- | --- | --- | --- | --- |
|  | Visit 1-2 | Visit 3 | Visit 4 | Visit 5 | Visit 6 | Visit 7 | Visit 8 | Visit 9 |  |
| IC | **○** |  |  |  |  |  |  |  |  |
| TdES |  | **①** | **②** | **③** | **④** | **⑤** | **⑥** |  |  |
| Blood pressure | **○** |  |  |  |  |  |  |  |  |
| Blood sampling | **○** |  |  |  |  |  |  |  |  |
| contrast-enhanced MRI | **○** |  |  |  |  |  |  |  |  |
| logMAR VA | **○** | **○** | **○** | **○** | **○** | **○** | **○** | **○** | **○** |
| ETDRS VA | **○** | **○** |  |  |  |  |  | **○** | **○** |
| HFA | **○** |  |  |  |  |  |  | **○** | **○** |
| Slit | **○** |  |  | **○** |  |  |  | **○** | **○** |
| IOP / fds | **○** |  |  | **○** |  |  |  | **○** | **○** |
| AEs | **○** | | | | | | | | |

**Abbreviations:** IC, informed consent; TdES, transdermal electrical stimulation; VA, visual acuity; HFA, Humphrey field analyzer; Slit, slit lamp examination; IOP, intraocular pressure; fds, fundus examination; AE, adverse event.

**Handling when an adverse event occurs**

Adverse events are all unwanted or unintended illnesses or disorders and their signs (clinical test values) that occur to the subject, user or others when using the study device, regardless of whether it has a causal relationship with the study device. Including abnormalities in). However, if it occurs in a person other than the subject, it is limited to the one suspected to be affected by the use of the investigational device.

Existing pathologies (history or complications that existed prior to the study period) will not be treated as adverse events unless they are exacerbated or the frequency of onset increases during the study period. However, if the complication worsens after the investigational device is performed, it will be treated as an adverse event, and the day when the deterioration is confirmed will be the date of occurrence of the adverse event.

A defect means that the condition of the investigational device is not good in terms of quality, safety, performance, etc., such as damage and malfunction, regardless of which stage of design, delivery, storage, or use. Information on defects in clinical trial equipment is hereinafter referred to as defect information.

Device-related adverse events are adverse events for which a causal relationship with the investigational device or procedure cannot be ruled out.

# If the investigator or investigator finds an adverse event or defect, he / she should take appropriate measures immediately, and if he / she discontinues the use of the investigational device or if treatment for the adverse event is required. Inform the subject to that effect. If an adverse event for which a causal relationship with the study device cannot be ruled out has not been recovered at the end or discontinuation of the study, in principle, observation will be continued as much as possible until recovery or improvement. However, this does not apply if the investigator or investigator determines that the effects of this study have disappeared, the safety of the subject is sufficiently ensured, and further follow-up is not necessary.

All adverse events that occur between the start of the study device and the end of the study will be reported regardless of whether there is a causal relationship with the study device, and the adverse events will be observed until the end of the study period (at the time of discontinuation). do. In addition, all adverse events and defect information that have been determined to have a causal relationship with the investigational equipment will be reported until the end of the clinical trial period.

The investigator or investigator shall describe all adverse events and defects that occurred during the above period in the medical record and case report form inconsistently.

**Contents required for evaluation of adverse events and defects**

1) Name of adverse event

As a general rule, the name of the adverse event should be recorded as the diagnosis name / disease name (disease name). If the diagnosis / disease name cannot be specified, or if the investigator or investigator determines that it is appropriate not to use the diagnosis / disease name, the clinical symptom or sign shall be the adverse event name.

2) Date of onset

3) Date of disappearance: Recovery, recovery or disappearance but with sequelae and date in case of death

4) Outcome: Recovery, remission, recovery or disappearance but with sequelae, unrecovered, dead, unknown

5) Treatment (enforcement of clinical trial equipment): No change, discontinuation, not applicable

6) Other treatments: None, drug treatment, etc.

7) Severity: Non-serious, serious

8) Severity: mild, moderate, severe

-Mild: Adverse events that are not considered to interfere with daily life. Example) To the extent that follow-up is possible

-Moderate: Adverse events that may interfere with daily activities. Example) Degree of need for some intervention or treatment

-Severe: Adverse events that may make daily life difficult. Example) To the extent that advanced and intensive treatment and systemic care are required

9) Causal relationship with investigational equipment: Association can be denied, association cannot be denied

1) Defect name

2) Defect confirmation date

3) Date of occurrence

4) Causes and status of problems that may have occurred

5) Measures for defects

6) Presence or absence of adverse events due to the defect

**Causal relationship between adverse event resilience and investigational equipment**

Recovery of adverse events means recovery to a state without adverse events or to a state before the implementation of the investigational device. When determining the causal relationship with the investigational device in an adverse event, the general condition of the subject, complications, concomitant drug / combination therapy, and temporal relationship should be taken into consideration.

・ Association can be denied: When the subject can be explained to be due to other factors before the start of clinical trial equipment or as shown in the following cases.

1) When the adverse event is a known side effect of the combination drug

2) The same adverse event does not occur even if the investigational device is re-performed.

・ Relationship cannot be denied: When the relationship with other factors cannot be clearly defined

**Treatment of investigational equipment**

1) No change: When an adverse event occurs, but there is no change in the conditions for conducting investigational device treatment.

2) Discontinuation: When the clinical trial device is discontinued due to the occurrence of adverse events

3) Weight loss: When the current intensity or duration of the investigational device is reduced due to the occurrence of adverse events.

4) Pause: When the operation of the investigational device is temporarily suspended due to the occurrence of an adverse event.

5) Enhancement: When the current intensity of the investigational device is enhanced or the duration is extended due to the occurrence of adverse events.

6) Not applicable: When an adverse event occurs before the start of clinical trial equipment or after the end of the clinical trial period

**Outcome**

1) Recovery: When the adverse event recovers or disappears to the state before the onset

2) Recovered but with sequelae: When the adverse event has recovered but the effects of the adverse event remain as sequelae

3) Death: When the adverse event that occurs is the direct cause of death

4) Relief: Adverse events continue, but symptoms tend to improve

5) Unrecovered: If the adverse event continues (symptoms do not tend to improve)

6) Unknown: When the subject cannot be tracked

**Definition of serious adverse events**

Serious adverse events shall fall under any of the following:

1) What leads to death (death)

2) Life-threatening (risk of death)

3) Items that require hospitalization or extension of hospitalization period

4) Permanent or significant disability / dysfunction

5) Those that cause birth defects

6) Other serious things according to the above

“Hospitalization” for the reasons described below is not considered a serious adverse event.

・ Includes re-examination, hospitalization for follow-up or extension of hospitalization period, and hospitalization (scheduled surgery, examination, etc.) for the sole purpose of performing treatment or examination scheduled before the start of the clinical trial during the clinical trial. No (however, new occurrences during the hospitalization are treated as adverse events).

・ Hospitalization for elective treatment for existing pathological conditions unrelated to the target disease of this study.

・ Hospitalization for social reasons and temporary rest of the caregiver without deterioration of general condition.

The subject of the report was all serious adverse events that occurred from the start of screening to the end (discontinuation) of the study, or events that may occur, and suspected relevance to the study equipment after the end (discontinuation) of the study. It is considered a serious adverse event.

**Procedures for reporting serious adverse events, etc.**

If an adverse event occurs and the investigator, etc. determines that it is serious, the adverse event information will be handled according to the following procedure.

1) Report from the investigator to the head of each medical institution

If the investigator finds that death or other serious adverse events have occurred or may occur due to a malfunction of the investigational equipment during the clinical trial period after the investigational equipment is conducted, the investigator immediately conducts each procedure. Report the content in writing to the head of the medical institution. When reporting, identify whether serious adverse events are unpredictable.

In addition, the investigator should describe the details of the event in the "Report on Serious Adverse Events and Malfunctions" (Unified Form 14) and "Form for Detailed Medical Description" as soon as possible, and the implementing medical institution. Report to the head of.

2) Report from the investigator to the clinical trial coordinating doctor and the sponsor of the clinical trial equipment

If the investigator finds that death or other serious adverse events have occurred or may occur due to a malfunction of the investigational equipment during the clinical trial period after the investigational equipment is conducted, the investigator immediately adjusts the clinical trial. Report the details to the doctor and the provider of the investigational device.

In addition, the investigator should describe the details of the event in the "Report on Serious Adverse Events and Defects" (common to Unified Form 14_ and Medical Details_Form 12_14_19) as soon as possible, and conduct the clinical trial. Report to the coordinating physician.

3) Consultation between the investigator and the investigator

The investigator shall consult with the investigator as necessary and report his / her opinion as the investigator (including the need to report to the Minister of Health, Labor and Welfare) to the investigation coordinating committee.

In addition, if the efficacy and safety evaluation committee is consulted regarding the judgment of the investigator, the opinions of the efficacy and safety evaluation committee will be followed.

4) Report to the Minister of Health, Labor and Welfare

If it is determined that the adverse event or defect is to be reported as stipulated in the Pharmaceuticals and Medical Devices Act, the clinical trial coordinating physician will prepare a "Medical Device Malfunction / Infectious Disease Case Report" (Attachment Form No. 8) and evaluate the efficacy and safety. Report to the committee and PMDA.

For the detailed procedure, follow the "Procedure Manual for Handling Safety Information (tentative name)".

5) Report to the director of the implementing medical institution

When a report is made to the Minister of Health, Labor and Welfare, the investigator shall obtain the "Medical Device Malfunction / Infectious Disease Case Report" (Attachment Form No. 8) obtained from the clinical trial coordinating doctor and, if necessary, "for medical details". Report the contents of the "Form" to the director of the implementing medical institution as soon as possible.

6) Response when additional information is obtained

When additional information about the event is obtained, the investigator of the conducting medical institution where the adverse event occurred shall make an additional report to the head of each conducting medical institution as soon as possible, and the study coordinating physician and the investigator. Report to the investigational device provider. The handling of the additional information shall be in accordance with the procedures 1) to 5) above, and shall be reported to PMDA as necessary.

**Case handling**

As a general rule, for registered cases, the investigator and the person in charge of statistical analysis will discuss and decide how to handle the cases. The clinical trial coordinator and the person in charge of statistical analysis will also decide on the handling of cases in the event of a new problem after consultation.

**Data handling**

As a general rule, the handling of data at the time of data aggregation / analysis shall be as shown below. If any doubt arises, the person in charge of statistical analysis and the investigator will make a decision before fixing the data in consultation.

Missing values are not complemented.

**Analysis of subject background**

Calculate the distribution and summary statistics of subject background data in each analysis target population. For nominal variables, the frequency and proportion of categories are shown. For continuous variables, calculate summary statistics.

**Safety and efficacy analysis**

Visual acuity (corrected decimal visual acuity converted to logMAR visual acuity is used to analyze the amount of change from baseline at week 12. A linear mixed-effects model is used as the statistical method, and the significance level of the hypothesis test is 5% on both sides. And the confidence interval is calculated as 95% confidence interval on both sides). If model analysis cannot be performed, only summary statistics are calculated. The detailed analysis method will be described in the statistical analysis plan.

Analysis of secondary efficacy endpoints included visual acuity (corrected decimal visual acuity converted to logMAR visual acuity and ETDRS visual acuity), static visual field test (HFA) 10-2 program and Estherman test (100 points). Perform statistical analysis. Multiplicity is not adjusted in the analysis of the secondary efficacy endpoint. A linear mixed-effects model is used as the statistical method, the significance level of the hypothesis test is 5% on both sides, and the confidence interval is 95% on both sides. If model analysis cannot be performed, only summary statistics are calculated. The detailed analysis method will be described in the statistical analysis plan.

For safety analysis, adverse events are aggregated, the number and proportion of cases of occurrence are aggregated, and a list is created for the presence or absence and severity of occurrence. In addition, the exact two-sided 95% confidence interval for the binomial distribution for the presence or absence of expression is calculated, and an appropriate significant difference test is performed as necessary.

**Effectiveness and Safety Evaluation Committee**

In this clinical trial, an efficacy and safety evaluation committee will be set up. The Efficacy and Safety Assessment Committee was established as an independent body of the investigator and consists of members of her three or more experts who are independent of the study. The Efficacy Safety Evaluation Committee conducts safety monitoring, such as comparison of adverse event incidence rates in study treatment and detailed examination of serious adverse events, as necessary, for the purpose of ensuring the safety of subjects. conduct. Occasionally, based on the results, in order to reduce the risk of adverse events, it may be recommended to change the clinical trial design such as changes in the inclusion criteria, or it may be decided whether or not the clinical trial can be continued. For details, refer to "Standard Business Procedures for Effectiveness and Safety Evaluation Committee".

**Final analysis**

After the follow-up period ends, data will be obtained and the case will be fixed before analysis. The person in charge of statistical analysis compiles an "analysis report" and submits it to the investigator and investigator. The clinical trial coordinator summarizes the contents of the analysis report, prepares a "summary report" that summarizes the conclusions, problems, interpretation and consideration of the results, future policies, etc. of the entire clinical trial from a clinical perspective, and is responsible for the clinical trial. Obtain the approval of a doctor.

**Compliance and deviation of clinical trial protocol**

1) The investigator or the investigator shall conduct the clinical trial in compliance with this clinical trial protocol.

2) If the investigator or the co-investigator deviates from the protocol, record the details and reasons for all deviations.

3) If the subject deviates from the implementation plan due to other medically unavoidable reasons in order to avoid an urgent crisis, the investigator immediately submits a document stating the details of the deviation and the reason to the director of the implementing medical institution. At the same time, the contents of the document will be promptly reported to the clinical trial review committee via the director of the implementing medical institution.

**Revision of clinical trial protocol and case report**

When revising the clinical trial protocol and case report, follow the procedure below.

1) When the investigator becomes aware of matters related to the quality, efficacy and safety of the investigational device, and other important information for conducting the clinical trial properly, the investigator will revise the investigational protocol as necessary. Also, when making revisions, create a revision history and save it.

2) The investigator will revise the case report as necessary in conjunction with the revision of the study protocol or for other reasons. Also, when making revisions, create a revision history and save it.

3) The investigator promptly submits the revised version of the clinical trial protocol and the revised case report form to the director of the conducting medical institution, and promptly obtains the approval of the clinical trial review committee via the director of the conducting medical institution.

4) The same procedure shall be applied when the clinical trial protocol and case report form are revised within the range allowed by the investigator to the extent that the director of the conducting medical institution gives instructions based on the opinion of the clinical trial review committee.

**Criteria for discontinuation or suspending of the entire clinical trial**

When the following information is available and it is considered difficult to continue the entire clinical trial, the investigator will discuss with the investigator and decide whether to discontinue or suspend the entire clinical trial.

1) When it becomes difficult to ensure the safety of the clinical trial due to new safety information about the clinical trial device or information on serious adverse events and defects.

2) When the implementing medical institution makes a serious violation of the GCP Ministerial Ordinance for medical equipment or a serious deviation from the clinical trial implementation plan.

3) In addition, when new information that may be necessary to discontinue or suspend the clinical trial is obtained during the clinical trial.

**Procedures for discontinuing or suspending the entire clinical trial**

If the clinical trial coordinator discontinues or suspends the entire clinical trial through consultation with other investigators or consultation with the Efficacy Safety Committee, etc., the director of the conducting medical institution and PMDA will be informed of the fact and the reason. Will be notified promptly in writing. In addition, the subject who is undergoing clinical trial equipment shall be promptly notified to that effect, and appropriate treatment such as change to appropriate treatment shall be performed.

**Procedures for discontinuing or suspending clinical trials at individual medical institutions**

If the investigator discontinues or suspend the clinical trial, the investigator will promptly notify the director of the conducting medical institution in writing and explain the discontinuation or suspending in detail in writing.

If the investigator notifies the investigator that the study has been discontinued or suspended, the investigator will promptly inform all investigators and PMDA involved in the study by means of a document detailing the discontinuation or suspending.

**End of clinical trial**

After the clinical trial is completed, the investigator will notify the director of the conducting medical institution in writing that the clinical trial has been completed, and will report the summary of the clinical trial results in writing.

**Data management procedure**

Detailed procedures for data management will be described in the data management plan.

**Data collection**

The investigator or investigator will prepare a case report using Electronic Data Capture (hereinafter referred to as “EDC”) that meets the requirements of 21 CFR Part 11, the GCP Ministerial Ordinance for Medical Devices, and the ER / ES Guidelines. The investigator or the investigator shall change, correct, or add to the contents of the case report on the EDC that created the case report, and record it as electronic information. In addition, if the investigator prepares a case report form by the investigator or if the investigator has transcribed the case report from the source material (original data), the investigator should submit the case report before the case report is submitted. Check the contents and confirm that there are no problems. The investigator will finally provide the electronic case report to the conducting medical institution in electronic media (eg, CD-R, etc.). The investigator ensures the readability and preservation of the electronic case report.

When using the EDC system, the implementing medical institution will take EDC training, and refer to the separate input manual for details on the input method.

**Identification of materials that should be entered directly in the case report form and should be understood as the source material (original data)**

In this clinical trial, the following documents, etc. will be used as the source material (original data).

1) Records related to the consent of the subject and the provision of information to the subject, medical records, nursing records, clinical test data, imaging test film, and other records that were the basis for creating case report reports. The data stored in the electronic medical record is also regarded as the source material.

2) Records regarding the implementation of clinical trial equipment

3) Documents or records related to the clinical trial necessary for the guidelines related to this clinical trial.

Of the data described in the case report form, the items shown below shall be the source material (original data) based on the description in the case report form. However, if there is a description in the medical record, etc., the medical record, etc. shall be regarded as the original material (original data).

1) Purpose of combination drug / combination therapy

2) Degree of adverse events, outcomes (including results at follow-up), severity, determination of causal relationship with study equipment and basis for determination

3) Reasons for discontinuing the clinical trial of the subject

4) Comments from the investigator or co-investigator

**Preservation of records by the implementing medical institution**

Medical Devices Documents or records related to clinical trials to be retained at the conducting medical institution specified by the GCP Ministerial Ordinance shall be retained by the hospital director for the period until the later of the following days.

1) Five years have passed from the date when the investigational device provider receives approval for manufacturing and marketing of the medical device related to the test device (if development is discontinued, three years from the date when development discontinuation is decided). Elapsed days)

2) The day when 3 years have passed since the trial was discontinued or completed.

The investigator will notify the conducting medical institution when it is no longer necessary to keep the records that should be kept by the investigating medical institution or the clinical trial review committee.

**Keeping records by the investigator**

Medical Devices Documents or records related to clinical trials to be retained by the investigator as stipulated in the GCP Ministerial Ordinance shall be retained in a storage location deemed appropriate for the period up to the later of the following.

1) Five years have passed from the date when the investigational device provider receives approval for manufacturing and marketing of the medical device related to the test device (if development is discontinued, three years have passed from the date when development discontinuation was decided. Day). However, for medical devices that must undergo re-examination after approval pursuant to the provisions of the Pharmaceutical Affairs Law and the period until the re-examination is completed exceeds 5 years, the date on which the re-examination will be completed.

2) The day when 3 years have passed since the trial was discontinued or completed.

**Direct viewing of the source material**

The head of the conducting medical institution and the investigator ensure that all records, including sources, are available for monitoring, auditing and investigations by the Trial Review Board or regulatory agencies. Make sure that the clinical trial is conducted properly and that the data is sufficiently reliable. The method of direct viewing and the timing of implementation will be specified separately in the monitoring plan.

**Quality management**

1) If there is an act that deviates from this protocol, the investigator or the co-investigator shall follow the provisions of this protocol.

2) The investigator or co-investigator prepares a case report in accordance with this study protocol.

3) The investigator confirms that all data and other records contained in the case report form are accurate and complete.

4) If any of the data contained in the case report is inconsistent with the source material, the investigator will create and keep a record explaining the reason.

5) The clinical trial coordinating physician will appoint a person who is not engaged in the clinical trial at the conducting medical institution subject to the monitoring as a monitor, and have him / her carry out monitoring according to the monitoring procedure manual reviewed by the clinical trial review committee. The monitor confirms the following items.

-The human rights, safety and welfare of the subject are protected.

-Medical devices GCP ministerial ordinance, the latest clinical trial implementation plan, and the procedure manual for the clinical trial are observed.

-The data reported by the investigator or the co-investigator will be accurate and complete, and will be verified against the clinical trial-related records such as the source materials.

6) The person in charge of data management formulates a data management plan in accordance with the standard business procedure manual separately established, and performs quality control at each stage of data handling to ensure the quality.

**Quality assurance**

The clinical trial coordinating doctor prepares a plan for auditing and a procedure manual for business, and based on the opinion of the clinical trial review committee, has the audit conducted in accordance with the plan and procedure manual. The person in charge of audit is not the person in charge of developing the clinical trial equipment related to the audit, and is also engaged in the implementation (including preparation and management) and monitoring of the clinical trial at the conducting medical institution related to the audit. Those who do not.

**Ethics and GCP**

This clinical trial will be conducted in accordance with the "Declaration of Helsinki (Revised 2013 Fortaleza General Assembly)", the Pharmaceutical Machinery Law and the GCP Ministry Ordinance for Medical Devices. In addition, it will be enforced in compliance with this clinical trial implementation plan and the procedure manual related to the clinical trial.

## In addition, when selecting a subject, the investigator or investigator depends on the subject's health condition, symptoms, age, gender, consent ability, investigator, etc. based on the viewpoint of human rights protection and selection criteria and exclusion criteria. Carefully consider the adequacy of requesting participation in clinical trials, taking into consideration the relationship and whether or not to participate in clinical trials including other clinical trials.

**Clinical Trial Review Committee**

Prior to conducting this clinical trial, the clinical trial review committee of the conducting medical institution will review the ethical, scientific and medical validity of this clinical trial. This clinical trial will be conducted after obtaining the approval of the clinical trial review committee. If the deliberation result of the clinical trial review committee is "approved after amendment", the implementation plan, case report, consent explanation document, etc. will be amended and approved based on the deliberation result, and then this clinical trial will be conducted. implement. In addition, the clinical trial review committee will continuously review whether or not this clinical trial is being conducted properly at least once a year.

**Health damage compensation and insurance**

If a subject suffers a health hazard as a result of participating in this clinical trial, the implementing medical institution will take necessary and appropriate measures such as providing a medical system for the treatment. However, in this clinical trial, medical expenses and medical allowances paid by the patient will not be paid. As a response to liability and compensation liability caused by health damage caused by this clinical trial, those involved in this clinical trial, such as investigators, investigators, conducting medical institutions, and clinical trial coordination committees, are covered by doctor-led clinical trial insurance (survivor compensation). Join money, funeral fees, disability compensation and disability compensation).

**Cost burden related to clinical trials**

The costs associated with this clinical trial are as follows.

1) The clinical trial equipment used in this clinical trial will be provided by Mayo Co., Ltd.

2) Expenses related to all examinations and diagnostic imaging during the clinical trial equipment period will be covered by non-insurance combined medical expenses.

3) Payment of the burden reduction fee for the subject shall be in accordance with the rules separately set by each implementing medical institution.

**Research funds and conflicts of interest**

This clinical trial will be conducted at the 2018 Chiba University Hospital Advanced Medical Development Promotion Expense (planned). Mayo Co., Ltd. provides information on clinical trial equipment under the GCP Ministerial Ordinance for Medical Devices, but is not involved in conducting, analyzing, or reporting clinical trials.

The interests of this clinical trial will be appropriately deliberated in accordance with the rules separately set by each conducting medical institution.

**Clinical trial database registration**

This clinical trial will be registered in the UMIN clinical trial registration system (http://www.umin.ac.jp/ctr/index-j.htm) before obtaining consent from the first subject.

# **References**

1. 三宅養三，他: EER（Electrically　Evoked　Response）の臨床応用Ⅳ視神経疾患のEER解析. 日眼会誌 **84**: 2047-52, 1980.
2. Morimoto T, et al: Electrical stimulation enhances the survival of axotomized retinal ganglion cells in vivo. Neuro Report **13**: 227-30, 2002.
3. Okazaki Y, et al: Parameters of optic nerve electrical stimulation affecting neuroprotection of axotomized retinal ganglion cells in adult rats. Neurosci Res **61**: 129-35, 2008.
4. Morimoto T, et al. Transcorneal electrical stimulation rescues axotomized retinal ganglion cells by activating endogenous retinal IGF-1 system. Invest Ophthalmol Vis Sci. 46:2147-2155, 2005.
5. Sato T, et al: Direct effect of electrical stimulation on induction of brain-derived neurotrophic factor from cultured retinal Muller cells. Invest Ophthalmol Vis Sci. 49: 4641-4646, 2008.
6. Ni YQ, et al.: Neuroprotective effect of transcorneal electrical stimulation on light-induced photoreceptor degeneration. Exp Neurol. 219; 2009:439-452.
7. Ciavatta VT, et al.: Growth factor expression following implantation of microphotodiode arrays in RCS rats. Invest Ophthalmol Vis Sci. 47:3177, 2006.
8. Zhou WT, et al.: Electrical stimulation ameliorates light-induced photoreceptor degeneration in vitro via suppressing the proinflammatory effect of microglia and enhancing the neurotrophic potential of Muller cells. Exp Neurol. 238:192-208, 2012.
9. Wang X, et al.: Neuroprotective effect of transcorneal electrical stimulation on ischemic damage in the rat retina. Exp Eye Res. 93:753-760, 2011.
10. Kanamoto T, et al.: Proteomic study of retinal proteins associated with transcorneal electric stimulation in rats. J Ophthalmol. 1-6, 2015.
11. Fu L, et al.: Transcorneal electrical stimulation inhibits retinal microglial activation and enhances retinal ganglion cell survival after acute ocular hypertensive injury. Transl Vis Sci Technol. 7:1-11, 2018.
12. Yin H, et al.: Transcorneal electrical stimulation promotes survival of retinal ganglion cells after optic nerve transection in rats accompanied by reduced microglial activation and TNF-α expression. Brain Res. 1650: 10-20, 2016.
13. Morimoto T, Fukui T, Matsushita K, Okawa Y, Shimojyo H, Kusaka S, Tano Y, Fujikado T: Evaluation of residual retinal function by pupillary constrictions and phosphenes using transcorneal electrical stimulation in patients with retinal degeneration. Graefes Arch Clin Exp Ophthalmol. 244:1283-1292, 2006.
14. Fujikado T, et al: Effect of transcorneal electrical stimulation in patients with nonarteritic ischemic optic neuropathy or traumatic optic neuropathy. Jpn J Ophthalmol. 50:266-73, 2006.
15. Schatz A, et al. Transcorneal Electrical Stimulation for Patients With Retinitis Pigmentosa: A Prospective, Randomized, Sham-Controlled Follow-up Study Over 1 Year. Invest Ophthalmol Vis Sci. 58: 257-69, 2017.
16. Sato T et al. Effect of Electrical Stimulation on IGF-1 Transcription by L-Type Calcium Channels in Cultured Retinal Müller Cells. Jpn J Ophthalmol. 52: 217-23, 2008.
17. Willmann G, et al. Gene Expression profiling of the retina after transcorneal electrical stimulation in wild-type brown Norway rats. Invest. Ophthalmol Vis Sci. 52: 7529-37. 2011.
18. Schatz A, et al. Transcorneal electrical stimulation for patients with retinitis pigmentosa: a prospective, randomized, sham-controlled exploratory study. Ophthalmol Vis Sci. 52: 485-96, 2011.
19. Bittner AK, et al. Longevity of visual improvements following transcorneal electrical stimulation and efficacy of retreatment in three individuals with retinitis pigmentosa. Graefes Arch Clin Exp Ophthalmol. 256: 299-306. 2018.
20. Inomata K, et al. Transcorneal electrical stimulationof retina to treat longstanding retinal artery occlusion. Graefes Arch Clin Exp Ophthalmol 245: 1773-80, 2007.
21. Oono S, et al Transcorneal electrical stimulation improves visual function in eyes with branch retinal artery occlusion. Clin Ophthalmol 5: 397-402. 2011.
