# Supplementary material for: Efficacy and safety of transdermal electrical stimulation in patients with nonarteritic anterior ischemic optic neuropathy
Source: Bioelectron Med. 2023 Oct 25;9:22. doi: 10.1186/s42234-023-00125-2 (PMC10598888; doi:10.1186/s42234-023-00125-2)
Supplement: Supplementary file 2 — Additional file 2. [file 42234_2023_125_MOESM2_ESM.docx]

**治験実施計画書**

**非動脈炎性虚血性視神経症を対象とした**

**経皮膚電気刺激の安全性及び有効性に関する試験**

**（第Ⅱ/Ⅲ相試験）**

| **版数** | **draft 2.0版** |
| --- | --- |
| **作成日** | **2018年11月30日** |

**改訂履歴**

| 作成日 | 版数 |
| --- | --- |
| 2018年10月11日 | draft 1.0版 |
| 2018年10月29日 | draft 1.1版 |
| 2018年11月12日 | draft 1.2版 |
| 2018年11月30日 | draft 2.0版 |
|  |  |

UMIN 試験ID：UMINXXXXXXXXX

**機密情報に関する注意**

　本治験実施計画書は機密情報であり、本治験に参加する治験責任医師、治験分担医師、治験協力者、実施医療機関、治験審査委員会、効果安全性評価委員会に対して提供されるものです。よって、被験者に説明する場合を除き、治験責任医師及び治験機器提供者である有限会社メイヨーの文書による同意なしに、いかなる第三者にも開示し、又は本治験の目的以外に利用することはできません。また、本治験の結果の一部又は全部を学会、雑誌等外部に発表する場合には、治験責任医師及び有限会社メイヨーの承諾が必要となりますので、あらかじめ、治験調整医師までご連絡頂けますようお願いします。

＜目次＞

[0. 治験の概要 8](#_Toc531591565)

[1. 緒言 11](#_Toc531591566)

[1.1. 治験の背景 11](#_Toc531591567)

[1.2. 非動脈炎性虚血性視神経症に対する標準治療 12](#_Toc531591568)

[1.3. 治験機器の概要 12](#_Toc531591569)

[1.3.1使用方法 12](#_Toc531591570)

[1.3.2. 非臨床成績 13](#_Toc531591571)

[1.3.3. 臨床試験成績 16](#_Toc531591572)

[1.3.4. 予想される有害事象及び不具合等 16](#_Toc531591573)

[2. 治験の目的と必要性及び経皮膚電気刺激治療の開発 17](#_Toc531591574)

[2.1. 治験の目的と必要性 17](#_Toc531591575)

[2.2. 臨床開発 17](#_Toc531591576)

[3. 対象患者 18](#_Toc531591577)

[3.1. 選択基準 18](#_Toc531591578)

[3.2. 除外基準 18](#_Toc531591579)

[4. 被験者の同意 19](#_Toc531591580)

[4.1. 同意文書及びその他の説明文書の作成並びに改訂 19](#_Toc531591581)

[4.2. 同意取得の時期と方法 19](#_Toc531591582)

[4.3. 被験者に対する説明事項 20](#_Toc531591583)

[5. 治験の方法 21](#_Toc531591584)

[5.1. 治験デザイン 21](#_Toc531591585)

[5.2. 目標被験者数と治験実施期間 22](#_Toc531591586)

[5.3. 施設登録・症例登録先 22](#_Toc531591587)

[5.3.1. 施設登録 22](#_Toc531591588)

[5.3.2. 症例登録 22](#_Toc531591589)

[5.3.3. 症例登録先 22](#_Toc531591590)

[5.4. 登録されなかった被験者の取り扱い 22](#_Toc531591591)

[5.5. 個々の症例の中止基準 22](#_Toc531591592)

[5.6. 併用可能薬及び併用可能療法 23](#_Toc531591593)

[5.7. 併用禁止薬及び併用禁止療法 23](#_Toc531591594)

[5.8. 併用制限薬 23](#_Toc531591595)

[5.9. 治験中止後の対応 23](#_Toc531591596)

[6. 治験機器 24](#_Toc531591597)

[6.1. 外観及び構成 24](#_Toc531591598)

[6.2 安全装置 25](#_Toc531591599)

[6.3. 作動原理 25](#_Toc531591600)

[6.4. 品質 25](#_Toc531591601)

[6.4.1. 安全性 25](#_Toc531591602)

[6.4.2. 性能 26](#_Toc531591603)

[6.4.3. 製造 26](#_Toc531591604)

[6.5. 包装・表示 27](#_Toc531591605)

[6.6. 管理方法 27](#_Toc531591606)

[7. 観察・検査・評価項目、方法及び実施時期 28](#_Toc531591607)

[7.1 実施スケジュールと手順 28](#_Toc531591608)

[7.1.1. スクリーニング検査 29](#_Toc531591609)

[7.1.2. 被験者の情報 29](#_Toc531591610)

[7.1.3. 観察・検査・評価項目 29](#_Toc531591611)

[7.1.3.1. 0週 30](#_Toc531591612)

[7.1.3.2. 2, 4, 8, 10週 30](#_Toc531591613)

[7.1.3.3. 6週 30](#_Toc531591614)

[7.1.3.4. 12週 31](#_Toc531591615)

[7.1.3.5. 中止時 31](#_Toc531591616)

[8. 有害事象発生時の取り扱い 32](#_Toc531591617)

[8.1. 有害事象、不具合及び機器関連有害事象の定義 32](#_Toc531591618)

[8.2. 有害事象又は不具合発生時の被験者への対応 32](#_Toc531591619)

[8.3. 報告の対象となる有害事象及び不具合 32](#_Toc531591620)

[8.4. 有害事象発生時の報告手順 32](#_Toc531591621)

[8.5. 有害事象の評価に必要な記載内容 32](#_Toc531591622)

[8.5.1. 有害事象 32](#_Toc531591623)

[8.5.2. 不具合 33](#_Toc531591624)

[8.5.3. 有害事象の回復性と治験機器との因果関係 33](#_Toc531591625)

[8.5.3.1. 治験機器との因果関係 33](#_Toc531591626)

[8.5.3.2. 治験機器に関する処置 33](#_Toc531591627)

[8.5.3.3. 転帰 34](#_Toc531591628)

[8.6. 重篤な有害事象発生時の取り扱い 34](#_Toc531591629)

[8.6.1. 重篤な有害事象の定義 34](#_Toc531591630)

[8.6.2. 報告の対象となる重篤な有害事象 34](#_Toc531591631)

[8.6.3. 重篤な有害事象の報告手順 34](#_Toc531591632)

[9. 評価項目 36](#_Toc531591633)

[9.1. 主要評価項目 36](#_Toc531591634)

[9.2. 副次評価項目 36](#_Toc531591635)

[9.3. 安全性評価項目 36](#_Toc531591636)

[10. 統計学的事項 37](#_Toc531591637)

[10.1. 解析対象集団 37](#_Toc531591638)

[10.1.1. 安全性解析対象集団 37](#_Toc531591639)

[10.1.2. 最大の解析対象集団 37](#_Toc531591640)

[10.1.3. 治験実施計画書に適合した対象集団 37](#_Toc531591641)

[10.2. 目標症例数と設定根拠 37](#_Toc531591642)

[10.3. 症例の取り扱い 37](#_Toc531591643)

[10.4. データの取り扱い 37](#_Toc531591644)

[10.5. 統計解析項目及び解析計画 38](#_Toc531591645)

[10.5.1. 被験者背景の解析 38](#_Toc531591646)

[10.5.2. 安全性及び有効性の解析 38](#_Toc531591647)

[10.5.2.1. 主たる解析 38](#_Toc531591648)

[10.5.2.2. 副次解析 38](#_Toc531591649)

[10.5.3. 安全性の解析 39](#_Toc531591650)

[10.6. 効果安全性評価委員会 39](#_Toc531591651)

[10.7. 最終解析 39](#_Toc531591652)

[11. 治験実施計画書の遵守及び逸脱 39](#_Toc531591653)

[12. 治験実施計画書、症例報告書又は解析計画に関する変更 39](#_Toc531591654)

[12.1. 治験実施計画書及び症例報告書の改訂 39](#_Toc531591655)

[12.2. 統計解析計画の変更 40](#_Toc531591656)

[13. 治験の中止、中断又は終了 40](#_Toc531591657)

[13.1. 治験全体での中止又は中断の基準 40](#_Toc531591658)

[13.2. 治験全体での中止又は中断する場合の手続き 40](#_Toc531591659)

[13.3. 個々の医療機関での治験の中止又は中断する場合の手続き 40](#_Toc531591660)

[13.4. 治験の終了 40](#_Toc531591661)

[14. データマネジメント 40](#_Toc531591662)

[14.1. データマネジメントの手順 40](#_Toc531591663)

[14.2. データの収集 41](#_Toc531591664)

[14.3. 症例報告書に直接記入され、かつ原資料（原データ）と解すべき資料の特定 41](#_Toc531591665)

[15. 記録の保存 41](#_Toc531591666)

[15.1. 実施医療機関による記録の保存 41](#_Toc531591667)

[15.2. 治験責任医師による記録の保存 42](#_Toc531591668)

[16. 原資料の直接閲覧 42](#_Toc531591669)

[17. 治験の品質管理及び品質保証 42](#_Toc531591670)

[17.1. 品質管理 42](#_Toc531591671)

[17.2. 品質保証 42](#_Toc531591672)

[18. 倫理及びGCP 43](#_Toc531591673)

[19. 治験審査委員会 43](#_Toc531591674)

[20. 健康被害補償及び保険 43](#_Toc531591675)

[21. 治験に関する費用負担 43](#_Toc531591676)

[22. 研究資金及び利益相反 43](#_Toc531591677)

[23. 治験のデータベース登録 44](#_Toc531591678)

[24. 治験実施体制 44](#_Toc531591679)

[25. 参考資料・文献リスト 45](#_Toc531591680)

＜略語・用語集＞

本治験実施計画書における略語・略記・用語の定義を以下に示す。

| 略号・略記・用語 | 定義 | |
| --- | --- | --- |
| AC | alternating current | 交流電流 |
| Ag/AgCl |  | 銀/塩化銀 |
| ALT | alanine aminotransferase | アラニンアミノ基転移酵素（GPT） |
| ALP | alkaline phosphatase | アルカリホスファターゼ |
| ANCA | anti-neutrophil cytoplasmic antibody | 抗好中球細胞質抗体 |
| AST | aspartate aminotransferase | アスパラギン酸アミノ基転移酵素（GOT） |
| BDNF | brain-derived neurotrophic factor | 脳由来神経栄養因子 |
| BUN | blood urea nitrogen | 尿素窒素 |
| CNTF | ciliary neurotrophic factor | 毛様体神経栄養因子 |
| COX-2 | cyclooxygenase-2 | シクロオキシゲナーゼ-2 |
| CRP | C-reactive protein | C反応性蛋白 |
| CRAO | central retinal artery occlusion | 網膜中心動脈閉塞症 |
| dB | decibel | デシベル |
| DC | direct current | 直流電流 |
| ERG | electroretinogram | 網膜電図 |
| ESR | erythrocyte sedimentation rate | 赤血球沈降速度 |
| ETDRS | early treatment diabetic retinopathy study | 早期糖尿病網膜症治療調査 |
| FAS | full analysis set | 最大の解析対象集団 |
| FGF1 | fibroblast growth factor | 線維芽細胞増殖因子1 |
| GOT | glutamic oxaloacetic transaminase | グルタミン酸オキサロ酢酸トランスアミナーゼ |
| GP | goldmann perimetry | ゴールドマン視野計 |
| HFA | humphrey field analyzer | ハンフリー視野計 |
| GPT | glutamic-pyruvic transaminase | グルタミン酸ピルビン酸トランスアミナーゼ |
| HbA1c | hemoglobin A1c | 糖化ヘモグロビン |
| Hz | hertz | ヘルツ |
| IGF-1 | insulin-like growth factors | インスリン様成長因子-1 |
| IL-1b | interleukin-1b | サイトカイン（IL-1b） |
| IL-6 | interleukin-6 | サイトカイン（IL-6） |
| IL-10 | interleukin-10 | サイトカイン（IL-10） |
| IS/OS | photoreceptor inner/outersegment junction | 視細胞内節外節接合部 |
| LD50 | lethal dose 50 | 半数致死量 |
| mA | milliampere | ミリアンペア |
| MD | mean deviation | 平均偏差 |
| mmHg | millimeter Hg | ミリメートル水銀柱 |
| msec | milli second | ミリ秒 |
| NAION | non-arteritic ischemic optic neuropathy | 非動脈炎性虚血性視神経症 |
| NGSP | national glycohemoglobin standardization program | 全米グリコヘモグロビン標準化プログラム |
| NF-κB | nuclear factor kappa-light-chain-enhancer of activated B cells | 転写因子 |
| PPS | per protocol set | 治験実施計画書に適合した対象集団 |
| RP | retinitis pigmentosa | 網膜色素変性 |
| TdES | transdermal electrical stimulation | 経皮膚電気刺激 |
| TES | transcorneal electrical stimulation | 経角膜電気刺激 |
| TNF-α | tumor necrosis factor-α | 腫瘍壊死因子 |

# 0. 治験の概要

| 治験課題名 | 非動脈炎性虚血性視神経症を対象とした経皮膚電気刺激の安全性及び有効性に関する試験 |
| --- | --- |
| 治験の目的 | 非動脈炎性虚血性視神経症患者に対する、皮膚電極を用いた経皮膚電気刺激による治療後の視機能への安全性及び有効性を確認すること。 |
| 治験デザイン | 単群、非盲検、多施設共同 |
| フェーズ | Ⅱ/Ⅲ相 |
| 被験機器 | 1. 概要   　電気刺激用皮膚電極を前額部の中央及び対象眼の下眼瞼耳側の皮膚に装着し、各種条件下である一定の電気刺激を与え、網膜及び視神経疾患等を対象として治療を行うための機器である。   1. 構成   　「電気刺激装置」、「皮膚電極」とそれらを繋ぐ「電極接続ケーブル」から構成されている。   1. 仕様 2. 電気刺激装置   ・刺激電流 0～3 mA  ・刺激時間 0～10 msec×2 biphasic  ・刺激間隔 50 msec   1. 皮膚電極　（積水化成品工業（株））   ・大きさ　　　　19×38 mmのディスポ電極  ・電極素子 Ag/Agcl  ・基材　　　 　 不織布  ・ゲル 　　　　導電性粘着ゲル   1. 電気容量   ACアダプタ定格（入力）　AC 100 V　50/60 Hz　30 VA  ACアダプタ定格（出力）　DC 5 V　4 A   1. 重量：約700 g、寸法： 225×165×40 mm |
| 選択基準 | 以下のすべての条件に該当する患者を対象とする。   1. 同意取得時の年齢が20歳以上80歳未満の非動脈炎性虚血性視神経症患患者。 2. 非動脈炎性虚血性視神経症と確定診断を受け、推定発症から6ヶ月以上経過し、症状固定と判断された患者。 3. 小数視力が手動弁以上0.7未満の患者。 4. 本治験の参加にあたり十分な説明を受けた後、十分な理解のうえ、本人の自由意思による文書同意が得られた患者。 5. 2週おきに12週間の通院が可能な患者。 |
| 除外基準 | 以下のいずれかの条件に該当する患者は対象としない。   1. 血液検査において、ESRが20 mm/h超、CRPが10 mg/L超の患者。 2. 非動脈炎性虚血性視神経症と確定診断を受けた時点で、造影MRIにより虚血性視神経症以外の眼病変が認められている患者。 3. 治験期間中に使用する予定の薬剤（散瞳薬、点眼麻酔薬等）に対し、薬剤アレルギーの既往のある患者。 4. 評価眼の視機能に重大な影響を及ぼす合併症を有する患者：硝子体黄斑牽引症候群／黄斑前膜／後部ぶどう腫を伴う強度近視等の黄斑病変、糖尿病網膜症、外眼部の炎症／感染症／重度のドライアイ、中等度以上の核白内障、視力に重大な影響を及ぼすような前嚢下／後嚢下／後発白内障。 5. 悪性腫瘍の既往又は合併のある患者。ただし、既往はあるが5年以上再発していない患者は登録可とする。 6. 認知症、精神疾患と診断され治療中の患者。 7. 血糖コントロールが著しく不良な糖尿病（HbA1c(NGSP) > 10.0%）を合併している患者。 8. 内服治療をしてもコントロール困難な高血圧症（収縮期≧180 mmHg、かつ／又は、拡張期≧110 mmHg）の患者。 9. スクリーニング時の臨床検査で、下記のいずれかに該当する肝・腎機能障害が認められる患者。  - AST、ALT：（施設）基準値上限の3倍超 - 血清クレアチニン：（施設）基準値上限の1.5倍超  1. エタンブトール塩酸塩及び／あるいはアオミドロン塩酸塩を服用中の患者。 2. 妊娠、授乳中（授乳を中止する場合も含む）又は本治験中に妊娠を希望している患者。 3. 現在、他の治験に参加している患者。 4. その他、治験責任医師又は治験分担医師が本治験の参加の対象として不適当と判断した患者。 |
| 評価項目 | ＜主要評価項目＞  logMAR視力（logMAR視力は矯正小数視力から換算）  ＜副次的評価項目＞   1. logMAR視力の0週からの変化量。 2. ETDRSチャートを用いたETDRS視力の0週から12週までの変化量。 3. 静的視野検査（HFA）：10-2 プログラムMD（Mean Deviation: MD）値の網膜感度のベースラインから12週までの変化量。 4. 静的視野検査（HFA）：エスターマンテスト（100点）スコアのベースラインから12週までの変化量。   ＜安全性評価項目＞  有害事象の発現状況（種類、頻度及び重症度等） |
| 治験方法 | 1. 本治験は、前観察期間及び治験期間から成る。   （詳細は、「7.1 実施スケジュールと手順」を参照する。）   1. 治験期間は12週間とし、皮膚電極を用いた経皮膚電気刺激治療を2週おきに6回施行する。本治療時の刺激条件は以下のとおりとする。   　　　＜刺激条件＞   - 電流強度：1.0 mA - パルス幅：10 ms/phase 双極性 - 刺激頻度：20 Hz - 刺激時間：30分 |
| 中止基準 | - 1. 被験者から治験参加辞退の申し出や同意撤回があった場合。   2. 治験登録後、被験者が対象として不適格であることが判明した場合。   3. 合併症の増悪により治験の継続が困難な場合。   4. 有害事象により治験の継続が困難な場合。   5. 妊娠が判明した場合。   6. 治験全体が中止された場合。   7. その他の重大な治験実施計画書違反が判明した場合。   8. 治験責任医師又は治験分担医師の判断により中止の必要性が認められた場合。 |
| 目標被験者数 | 5例5眼 |
| 治験実施期間 | 治験実施期間： 1年3ヵ月 （2019年4月1日～2020年6月30日）  症例登録期間： 1年 　　　（2019年4月1日～2020年3月31日） |
| 治験施設数 | 3施設（予定） |
| 倫理及びGCP | 本治験の実施に際しては「ヘルシンキ宣言」に基づく倫理的原則、「医薬品、医療機器等の品質、有効性及び安全性の確保等に関する法律（以下、「薬機法」という。）」、「医療機器の臨床試験の実施の基準に関する省令（以下、「医療機器GCP省令」という。）」及びその他の関連する規制要件を遵守するものとする。 |
| 治験審査委員会 | 本治験の実施に先立ち、実施医療機関の治験審査委員会は、本治験の倫理的、科学的及び医学的妥当性を審査する。本治験は、治験審査委員会の承認を得た後に実施する。治験審査委員会の審議結果が「修正の上で承認する」であった場合には、審議結果に基づいて実施計画書又は症例報告書、同意説明文書等を修正した後、本治験を実施する。また、治験審査委員会は少なくとも1年に1回以上の頻度で本治験が適切に実施されているか否かを継続的に審査する。 |

# 1. 緒言

## 1.1. 治験の背景

非動脈炎性虚血性視神経症（以下、「NAION」という。）は、後毛様体動脈のアテローム動脈硬化による閉塞又は狭小化によって視神経における急性の虚血により視機能障害が生じる疾患である。視神経の虚血により視神経乳頭色調の蒼白化及び周囲に綿花状白斑や火焔状の網膜出血を認める。典型的には比較的高齢の50歳以上の患者にみられ、欧米では50歳以上の10万人に対して2～10人程度の発症と言われている。多くは片眼性に突然、視力障害、視野障害が生じ、水平性視野欠損が典型的だが、中心視野欠損を示す場合もある。視神経の強膜篩板の手前で虚血を起こす、前部虚血性視神経症が多数を占める。1～数日で症状の固定が起こり、発症から4～8週後に視神経萎縮となるが、萎縮はしばしば分節状に生じる。視神経における陥凹乳頭比が小さいことがNAIONの危険因子とされているが、通常、非動脈炎型の明らかな原因とされる病態は見つかっていない。一部の患者は動脈硬化に寄与する因子（例：糖尿病、喫煙、高血圧、肥満）、閉塞性睡眠時無呼吸症、特定の薬物（例：アミオダロン、場合によりホスホジエステラーゼ-5阻害薬）、及び凝固亢進疾患を有しており、これらが危険因子と考えられている。稀に、抗リン脂質抗体症候群や、ANCA関連血管炎を合併している場合がある。起床時に視力障害がみられることから，非動脈炎型の潜在的原因として夜間の低血圧が疑われている。また20 %前後の症例において僚眼に発症することもあると報告されている。

現在確立した有効な治療法は存在しない。もう一方のNAIONを発症していない健康な眼に発症することもあるため、予防療法として抗凝固療法、血管拡張療法、ステロイド内服などが選択されることもあるが有意な視機能改善のエビデンスを得るまでには至っておらず、一般的な治療法としては普及していない。

電気刺激が網膜神経節細胞に対して神経保護効果を有するとの基礎研究結果 ^1, 2, 3^が報告されている。網膜電気刺激を行うことによって、網膜内層におけるIGF-1の活性上昇^4^、BDNF（脳由来神経栄養因子）の発現増加^８^、CNTF（毛様体神経栄養因子）発現増加^６^、FGF1の発現増加^７^、IL-1b及びTNF-αの抑制^８^、Bax遺伝子のダウンレギュレーション^６^、グルタミン合成酵素分泌増加^９^、細胞内アデノシン1リン酸レベルの増加^２^、細胞シグナル伝達・代謝・構造・免疫学的因子関連タンパク質のアップレギュレーション^10^、IL-10発現増加に伴うIL-6・COX-2発現減少^11^、NF-κBリン酸化減少^11^、小膠細胞由来のTNF-α産生抑制^12^、脈絡膜血流増加^13^等、様々な機序によって網膜神経保護及び機能活性化を生じると報告されている。

また臨床においても、虚血性視神経症患者に対して網膜電気刺激を行うことによって視力、中心フリッカー値、視野の改善を認めたとの報告^14^が存在する。

NAIONの病態は眼動脈の分枝である後毛様体動脈の閉塞による視神経および神経節細胞を含む網膜内層障害であり、上述の機序による神経保護及び生存細胞の賦活化が期待できる網膜電気刺激療法は、NAIONにおいても視機能の改善が期待される治療法であると考えられる。

海外では既に小型のポータブル型の器械が商品化され、主に欧州ではRPや加齢黄斑変性等の網膜疾患を対象として、眼瞼を覆う皮膚電極と手の甲の皮膚電極を用いた電気刺激装置である“ScyFix^®^”や、コンタクトレンズ型角膜電極を用いた電気刺激装置である“OkuStim^®^”が臨床に用いられているものの、国内では商品化されていない。また、TESの“OkuStim^®^”は、角膜上に電極を設置して電気刺激を行うため、操作は簡便ではあるが、ドライアイの有害事象が報告^15^され、電気刺激による角膜障害の危険性が指摘されている。それに対して、本研究で用いる電気刺激治療は、電極を両頬と額の皮膚に設置することで、角膜電極と比較しより低侵襲に、より簡便に電気刺激を行うことが出来ると考える。

千葉大学医学部附属病院眼科において、RP患者10例20眼を対象に2週間隔で6回の両頬と額に設置する皮膚電極を用いた経皮膚電気刺激（以下、「TdES」という。）治療を実施した「網膜色素変性を対象とした経皮膚電気刺激の安全性および有効性を検討する探索的試験（第Ⅱ相試験）」において、重篤な有害事象の発現及び不具合等の発生はなかった。有害事象が4例（感冒4例、下痢1例、発熱1例）に発現したが、治験機器との因果関係は認められなかった。一方、有効性の検討においては、ETDRS視力が6週、10週及び12週時において0週時に比べ統計学的に有意な改善を示し、12週時では平均で約4文字の改善を認めた。logMAR視力においても8週時以降、0週時に比べ統計学的に有意な改善を示した。静的視野検査でのMean Deviation値（以下、「MD値」という。）は改善傾向であり、12週時において、0週時との統計学的な有意差を認めた。

上記治験成績により、安全性が確認されたこと、および疾患は異なるが視機能の改善効果が認められたことおよび網膜電気刺激の機序から、NAIONにおいてもTdESによる神経保護効果によって視力及び視野が改善する可能性が考えられる。TdESは侵襲性が低く、経済的にも負担の少ない治療法となる可能性があり、臨床上有用であると考えられることから、皮膚電極を用いた本治療法の開発を検討するに至った。

以上より、現状において確立された治療方法が存在せず、一旦発症すると重度の視神経障害による視野障害を来し生活の質を著しく低下させるNAIONにおいて、視力および視野の改善が期待できるTdESの効果を調査することは大きな意義があると考えられ、本治験を計画した。

## 1.2. 非動脈炎性虚血性視神経症に対する標準治療

　NAIONの治療については，視力改善に関して現時点では明らかな効果のあるものはなく、治療法の確立は重要な問題である。

## 1.3. 治験機器の概要

### 1.3.1使用方法

電気刺激の強さ（電流量）及び時間（治療時間）をコントロールするほか、治療眼（右眼、左眼、両眼）の選択を行う。本体には、電気刺激で通電する電流の大きさ、通電時間等を表示する。皮膚電極はゲル状の粘着物質がある面を皮膚に接触し、反対の面にある突起に皮膚電極と本体を接続するケーブルを接続する。電極の装着場所は、治療眼の下眼瞼耳側及び前額部の中央に装着する。

| ゲル状パッドの電極は、家庭向け低周波治療器に使用されるディスポーザブルな電極に類似する。大きさは19×38 mm程度で、ゲル状の粘着物質がある面を皮膚に接触し、反対の面にある突起に電極と機器を接続するケーブルを接続し、皮膚を経由して電気刺激が行われる。電極の装着場所は、治療眼の下眼瞼耳側及び前額部の中央に装着する（右図）。 | 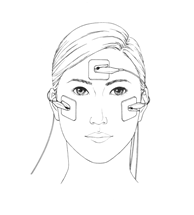  電極装着図 |
| --- | --- |

### 1.3.2. 非臨床成績

当該機器にしようする電極パッドが健常な皮膚に接触することから、生物学的安全性について担保が求められる。電極パッドの製造業者である積水化成工業株式会社の生物学的試験データによると、生体用ハイドロゲル（テクノゲル^®^）には、生体安全性関連の有害事象はないとされており、心電図用電極、脳波用電極、生体センサー、電気治療器用電極などに使用されている。試験データの概要を以下に記す。

【生物学的試験データ（SRA240/80-09S）】

1. 以下の規格に基づいて生物学的試験が実施された。

| 試験 | 規格及び試験方法 |
| --- | --- |
| 皮膚一次刺激性試験 | ISO 10993-10:2010 - 医療機器の生物学的評価 Part 10: 刺激性及び遅発性過敏性の試験 (sectiuon 6.3) |
| 感作性試験 | ISO 10993-10:2010 - 医療機器の生物学的評価 Part 10: 刺激性及び遅発性過敏性の試験 (sectiuon 7.2)  ISO 10993-12:2012 - 医療機器の生物学的評価 Part 12: 試料調製及び対象物質 |
| 細胞毒性試験 | ISO 10993-5:2009 - 医療機器の生物学的評価 Part 5: 細胞毒性試験: in vitro法 |
| 河合法  「24時間開放性塗布試験」 | 被験者20名の上腕内側部に試料を24時間塗布し、塗布位置を観察する。肉眼的に異常のないものはレプリカ標本を作製し全視野を顕微鏡観察し、刺激性の有無及び強度を判定する。 |

1. 結果概要 （SRA240/80-09S）

| Test Method | Result |
| --- | --- |
| 細胞毒性試験　非接触（寒天）法 | 細胞毒性なし |
| マウスを用いた局所リンバ節アッセイ | 陰性 |
| ウサギを用いた皮膚1次刺激性試験 | P.I.I=0, 刺激性なしe |
| 河合法「24時間開放性塗布試験」 | 3B-0C-0D、ヒトに対する刺激性は極めて弱い |

　NAIONに対する治療法は確立されていないため、新たな治療法が模索されており、下記に網膜電気刺激に関する基礎研究の報告がある。

| 文献 | 実験系 | 電気刺激経路 | 刺激条件 | 結果 |
| --- | --- | --- | --- | --- |
| Sato T 他  (2008) ^16^ | 1継代培養ミュラー細胞（生後12-14日のLong-Evansラット） | In vitro | パルス1ms, 20Hz, 0, 1, 5. 10 mA, 30分 | ミュラー細胞内IGF-1 mRNA増加  L型Ca^2+^チャネルからのCa^2+^細胞内流入増加 |
| Sato T他  (2008)^5^ | 1継代培養ミュラー細胞（生後12-14日のLong-Evansラット） | In vitro | パルス1ms, 20Hz, 10 mA, 30分 | ミュラー細胞内脳由来神経栄養因子（BDNF）mRNA増加  ミュラー細胞内BDNF増加 |
| Morimoto T他  (2002)^2^ | 視神経切除Wistar雄ラット | 視神経切断端 | 20Hz, 20, 30, 50, 70 μA, 2時間 | 網膜神経節細胞（RGC）に対する神経保護効果網（RGC生存率が電流強度に応じ増加） |
| Morimoto T他  (2005)^4^ | 視神経切除Slc:Wistar雄ラット | 経角膜 | 双極パルス20Hz, 100μA, 1時間 | 膜神経節細胞（RGC）の生存率増加,  網膜内ミュラー細胞でのIGF-1産生増加 |
| Willmann G 他  (2011)^17^ | Brown Norwayラット | 経角膜 | 双極パルス 1-ms, 20 Hz, 200 μA, 1時間 | 網膜細胞のアポトシスと関連するBax及び仕様壊死因子（TNF）ファミリー（Tnfrsf11b, Tnrsf12a, Tnrsf13b, Tnrsf13）のダウンレギレーション（発現抑制） |

### 1.3.3. 臨床試験成績

国内では実績がなく、海外では、ドイツがOkuvision社製OkuStim^®^を、アメリカがScyFix社製ScyFix700^®^を電気刺激治療器として販売している。文献の要旨を以下に示す。

| Schatz A 他^18^ | RP24例にTES　週1回30分6回、安全性及び忍容性に問題なし、有効性：視野と暗順応b波が有意に改善。 |
| --- | --- |
| Schatz A他^15^ | RP52例、週1回30分1年間、安全性結果：安先生：一過性ドライアイ症状（52人中31人）、他に臨床的に有意な有害事象なし。有効性結果：明順応b波が有意に改善、暗順応b波振幅が改善傾向。 |
| Bittner AK 他^19^ | 2.4〜3年にわたりTESを3〜6回受けた網膜色素変性患者3人1、安全性：治療と関連ある有害事象なし、有効性：中心視機能の改善をTES後約4〜7週間後に繰り返し認め、その後ベースラインに回帰し、かつベースラインを超えて視機能が大幅に低下することはなかった。 |
| Inomata K他^20^ | 網膜中心動脈閉塞症（以下、「CRAO」という。）2眼、BRAO1眼、VA及びmfERGは2例で改善し、3例すべでVFが改善した。 |
| Oono S 他^21^ | BRAO長期2 眼、初診 3眼、有効性；VA2眼に有意に改善、変化なし1眼、1眼に、P1の潜時有意に短縮。 |
| Fujikado T 他^14^ | NAION3例、TON（外傷性視神経症）5例 、NAION2例及びTON4例において視力の改善を認めた。 |

### 1.3.4. 予想される有害事象及び不具合等

使用する電流量からは有害事象が発生する可能性は低いと推測されるが、電極装着部位に一致した皮膚炎や角膜障害が生じる可能性がある。また電気刺激による電極装着部位付近の皮膚に刺激感を感じることが予測される。医療機器の不具合情報に加えて、不適切な使用方法（未熟な手技）による不具合情報も含める。

# 2. 治験の目的と必要性及び経皮膚電気刺激治療の開発

## 2.1. 治験の目的と必要性

NAIONには標準治療法がないことから、NAION患者に対する、皮膚電極を用いた経皮膚電気刺激による治療後の視機能への有効性及び安全性を確認することは臨床上必要と考えられる。

## 2.2. 臨床開発

TdESを用いたRPの10例20眼を対象とした第Ⅱ相試験は、2018年2月に終了し安全性が示唆された。有効性の評価項目であるlogMAR視力、ETDRS視力、静的視野検査（HFA）10-2プログラムのMD値が統計的に有意（p < 0.05）な改善を示した。

TdESを用いたRPの第Ⅲ相治験は現在計画中であり、20例20眼で実施予定である。RPは厚生労働省の難病指定を受けている希少疾患であり、両治験において計30例の有効性及び安全性が評価される。2017年に実施した独立行政法人医薬品医療機器総合機構（以下、「PMDA」という。）とのレギュラトリーサイエンス（以下、「RS」という。）戦略相談時に、TdESを用いたNAION及びCAROも対象に開発することが推奨された。NAION及びCRAOはウルトラオーファン（欧州医薬品庁の定義：患者数が5万人に1人未満）であることから、これらの疾患に関する治験を単独で実施することは困難であり、5~10例程度の被験者での効果の確認が必要である。このことから、

RP、CRAO及びNAIONを対象とした各々のTdESを用いた治験は、医薬品における希少疾病及びウルトラオーファンにおける国内での承認申請時に評価された日本人被検者数は40例以下の場合もあり、医療機器であるTdES治療の日本人被験者総計は40例と少数ではあるが承認申請するに妥当な症例数と考えられる。

# 3. 対象患者

以下の選択基準のすべてを満たし、除外基準のいずれにも該当しない患者を治験登録適格例とする。

## 3.1. 選択基準

以下のすべての条件に該当する患者を対象とする。

1. 同意取得時の年齢が20歳以上80歳未満の非動脈炎性虚血性視神経症患者。
2. 非動脈炎性虚血性視神経症と確定診断を受け、推定発症から6ヶ月以上経過し、症状固定と判断された患者。
3. 小数視力が手動弁以上0.7未満の患者
4. 本治験の参加にあたり十分な説明を受けた後、十分な理解のうえ、本人の自由意思による文書同意が得られた患者。
5. 2週おきに12週間の通院が可能な患者。

| 【設定根拠】  1, 2, 3) NAIONは視力悪化が固定する疾患であり、治験機器の性能が検出可能な、視力が中等度～重度の間の中間層の患者を対象とするため。  4) 「ヘルシンキ宣言」に基づく倫理的原則、薬機法、医療機器GCP省令及びその他の関連する規制要件を遵守して治験を実施するため。  5) 本治験を完遂出来ると考えられる患者を対象とするため。 |
| --- |

## 3.2. 除外基準

以下のいずれかの条件に該当する患者は対象としない。

1. 血液検査において、ESRが20 mm/h超、CRPが10 mg/L超の患者。
2. 非動脈炎性虚血性視神経症と確定診断を受けた時点で、造影MRIにより虚血性視神経症以外の眼病変が認められている患者。
3. 治験期間中に使用する予定の薬剤（散瞳薬、点眼麻酔薬等）に対し、薬剤アレルギーの既往のある患者。
4. 評価眼の視機能に重大な影響を及ぼす合併症を有する患者：硝子体黄斑牽引症候群／黄斑前膜／後部ぶどう腫を伴う強度近視等の黄斑病変、糖尿病網膜症、外眼部の炎症／感染症／重度のドライアイ、中等度以上の核白内障、視力に重大な影響を及ぼすような前嚢下／後嚢下／後発白内障。
5. 悪性腫瘍の既往又は合併のある患者。ただし、既往はあるが5年以上再発していない患者は登録可とする。
6. 認知症、精神疾患と診断され治療中の患者。
7. 血糖コントロールが著しく不良な糖尿病（HbA1c(NGSP) > 10.0%）を合併している患者。
8. 内服治療をしてもコントロール困難な高血圧症（収縮期≧180 mmHg、かつ／又は、拡張期≧110 mmHg）の患者。
9. スクリーニング時の臨床検査で、下記のいずれかに該当する肝・腎機能障害が認められる患者。

- AST、ALT：（施設）基準値上限の3倍超
- 血清クレアチニン：（施設）基準値上限の1.5倍超

1. エタンブトール塩酸塩及び／あるいはアオミドロン塩酸塩を服用中の患者。
2. 妊娠、授乳中（授乳を中止する場合も含む）又は本治験中に妊娠を希望している患者。
3. 現在、他の治験に参加している患者。
4. その他、治験責任医師又は治験分担医師が本治験の参加の対象として不適当と判断した患者。

| 【設定の根拠】  1, 2) 類似疾患の動脈炎性虚血性視神経症との鑑別のため。  3, 5, 6, 7, 8, 9, 10, 11) 対象患者の安全性を考慮するため。  4, 12, 13) 対象患者の正確な有効性を評価するため。 |
| --- |

# 4. 被験者の同意

## 4.1. 同意文書及びその他の説明文書の作成並びに改訂

治験責任医師は、被験者から治験参加の同意を得るために用いる同意文書及びその他の説明文書を可能な限り平易な表現で作成する。また、同意文書及びその他の説明文書を改訂する必要があると認めた場合は、これらを改訂する。

治験責任医師は、作成又は改訂された同意文書及びその他の説明文書を治験審査委員会に提出し、その承認を得る。

## 4.2. 同意取得の時期と方法

1. 同意の取得

治験責任医師又は治験分担医師は、治験審査委員会の承認を得た同意文書及びその他の説明文書を被験者に手渡し、「4.3 被験者に対する説明事項」に示す内容について十分な説明を行う。また、必要な場合には、治験協力者も被験者に補足的な説明を行う。被験者が治験の内容を良く理解したことを確認した上で、前観察期間（スクリーニング）検査を実施するまでに文書で自由意思による同意を取得する。

1. 説明時の被験者への対応

治験責任医師又は治験分担医師は、同意を得る前に被験者が質問をする機会と当該治験に参加するか否かを判断するのに十分な時間を与え、被験者の質問に対しては、被験者が満足するように回答する。

1. 同意書への記入方法及び説明文書の交付

被験者の同意に際しては、説明を行った治験責任医師又は治験分担医師が記名押印又は署名し、説明した日付を記入する。被験者は同意書に記名押印又は署名し、同意した日付を記入する。なお、治験協力者が補足的な説明を行った場合は、当該治験協力者も記名押印又は署名し、説明した日付を記入する。同意を得た後、説明文書及び同意書の写しを被験者に交付する。

1. 説明文書改訂時

治験責任医師又は治験分担医師は、被験者の同意に関連し得る新たな情報の入手等により同意文書及びその他の説明文書を改訂した場合、その都度、被験者に対して改訂された同意文書及びその他の説明文書を用いて改めて説明し、治験への参加継続について被験者から文書で自由意思による再同意を取得する。なお、被験者の同意に影響を与えうる新たな重要な情報が得られた場合には、直ちに当該情報を被験者に提供し、これを文書により記録するとともに、被験者が治験に継続して参加するかどうかを確認する。

## 4.3. 被験者に対する説明事項

治験責任医師が作成する説明文書には、以下の事項を記載する。

1. 当該治験が研究を伴うこと
2. 治験の目的
3. 治験責任医師の氏名、職名及び連絡先
4. 治験の方法
5. 予期される臨床上の利益及び危険性又は不便
6. 被験者に対する他の治療方法の有無及びその治療方法に関して予測される重要な利益及び危険性
7. 被験者の治験に参加する予定期間
8. 治験への参加は被験者の自由意思によるものであり、被験者は治験への参加を随時拒否又は撤回することができること。また、拒否・撤回によって被験者が不利な扱いを受けることはないこと、治験に参加しない場合にうけるべき利益を失うことはないこと
9. モニター、監査担当者、治験審査委員会及び規制当局が医療に係る原資料を閲覧できること。 その際、被験者の秘密は保全されること。
10. 治験の結果が公表される場合であっても、被験者の秘密は保全されること
11. 治験に関連する健康被害が発生した場合に被験者が受けることのできる補償及び治療
12. 治験への参加継続について被験者の意思に影響を与える可能性のある情報が得られた場合には速やかに被験者に伝えられること
13. 治験への参加を中止させる場合の条件又は理由
14. 治験に関し被験者が負担をする費用
15. 治験に関し被験者に金銭等が支払われる場合にはその内容（支払額算定の取決め等）
16. 被験者が治験及び被験者の権利に関してさらに情報がほしい場合又は治験に関連する健康被害が生じた場合に照会すべき又は連絡をとるべき医療機関の相談窓口
17. 被験者が守るべき事項
18. 当該治験の適否等について調査審議を行う治験審査委員会の種類、各治験審査委員会において調査審議を行う事項その他当該治験に係る治験審査委員会に関する事項
19. 知的財産
20. 利益相反

# 5. 治験の方法

## 5.1. 治験デザイン

本治験は、NAION患者に対する、皮膚電極を用いた経皮膚電気刺激による治療後の視機能への安全性及び有効性を確認する非盲検非対照第Ⅱ/Ⅲ相試験である。

　本治験のデザインは以下の通りである。


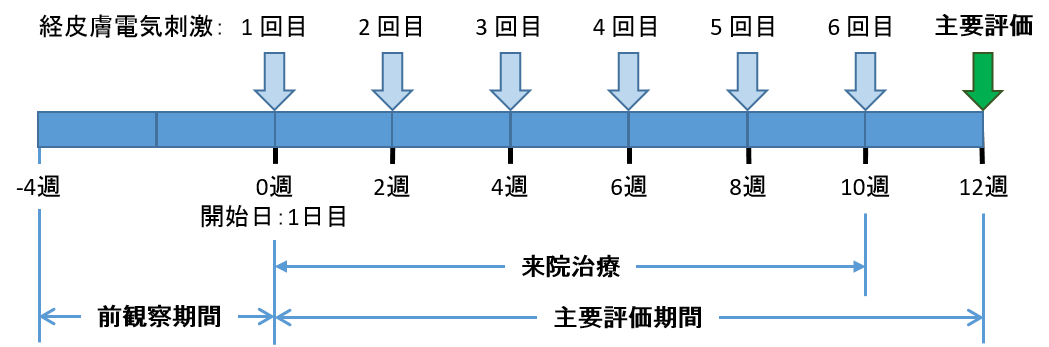


＜刺激条件＞

- 電流強度：1.0 mA
- パルス幅：10 ms/phase 双極性
- 刺激頻度：20 Hz
- 刺激時間：30 分

【刺激条件の設定根拠】

刺激条件は「網膜色素変性を対象とした経皮膚電気刺激の安全性および有効性を検討する探索的試験（第Ⅱ相試験）」において安全性が示唆され、logMAR視力、ETDRS視力及び静的視野検査で有効性の認められた条件と同じに設定した。

＜刺激方法＞


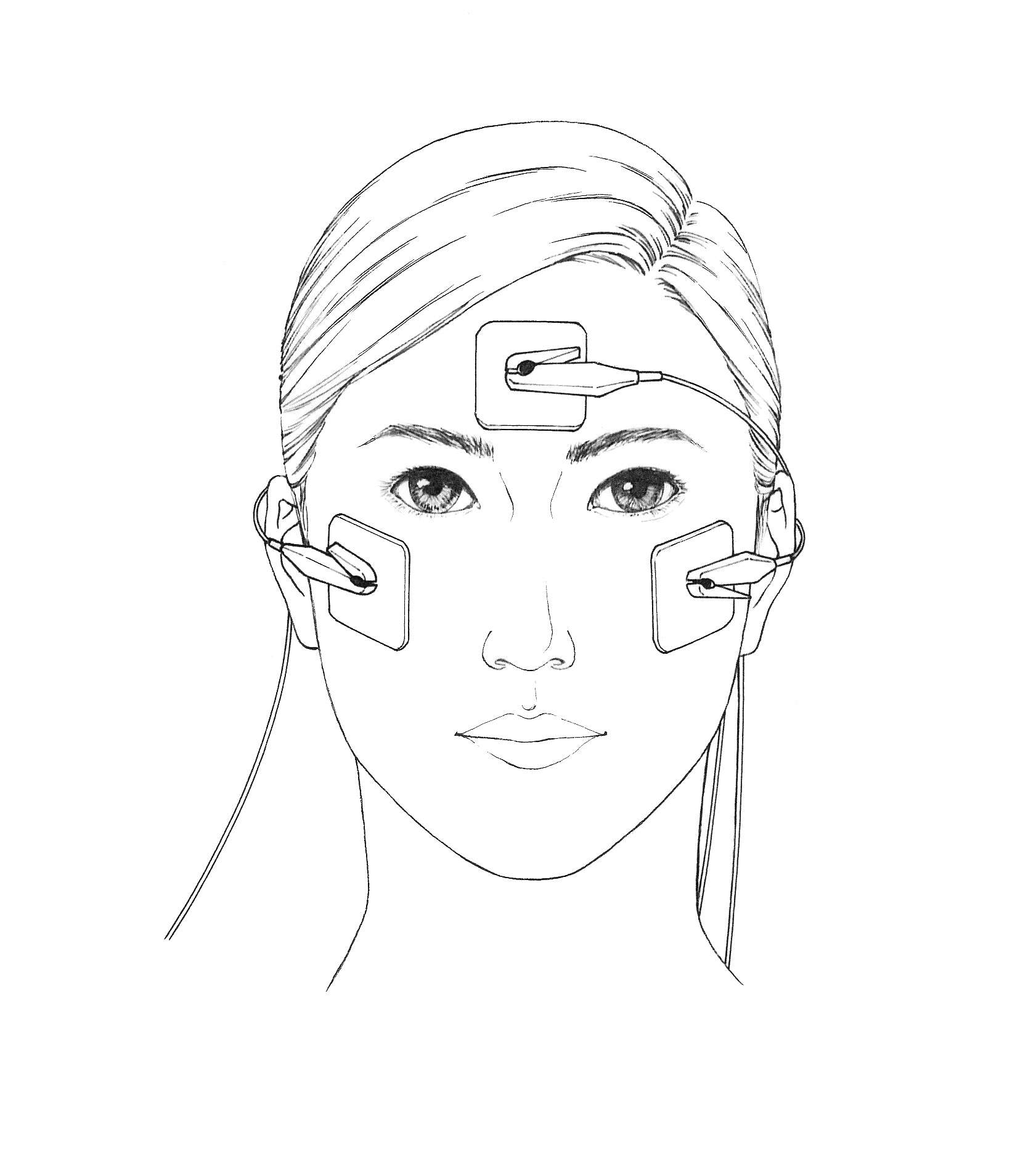


対象患者の前額部の中央及び対象眼の下眼瞼耳側の皮膚に電極をつけて、電気刺激装置から電極を通して電気刺激を行う（左図参照）。

TdES実施日を記載する。

## 5.2. 目標被験者数と治験実施期間

目標被験者数： 5例5眼

治験実施期間： 1年3ヵ月 （2019年4月1日～2020年6月30日）

症例登録期間： 1年 　　　（2019年4月1日～2020年3月31日）

## 5.3. 施設登録・症例登録先

施設登録及び症例登録は、千葉大学医学部附属病院 臨床試験部 データマネジメント室における中央登録制とする。

### 5.3.1. 施設登録

1. 治験責任医師は、当該施設の治験審査委員会で承認が得られた後、治験審査委員会の承認通知書の写し及び施設登録依頼書を症例登録センターにFAX する。
2. 症例登録センターは施設登録を行い、施設登録完了通知書を治験責任医師に送付する。

### 5.3.2. 症例登録

1. 治験責任医師又は治験分担医師は、文書による同意を取得し、スクリーニング検査の結果、被験者が選択基準を満たし、除外基準に抵触していないことを確認する。治験責任医師又は治験分担医師が「適格」と判断した被験者について、治験責任医師、治験分担医師又は治験協力者は症例登録を行う。症例登録は、Webサイトにより行う。
2. 治験責任医師、治験分担医師又は治験協力者は指定されたURL にアクセスし、Webサイト上で症例登録に必要な情報を入力する。治験責任医師又は治験分担医師は適格性判定を画面上で確認し、適格と判定された場合、プロトコル治療を開始する。一度登録された被験者の登録取り消しはされない。重複登録の場合は、いかなる場合も初回の登録情報(登録番号)を採用する。誤登録・重複登録が判明した際には速やかに千葉大学医学部附属病院 臨床試験部 データマネジメント室に連絡する。

※ 治験責任医師又は治験分担医師は、被験者の登録がなされるまでプロトコル治療をしてはならない。

### 5.3.3. 症例登録先

千葉大学医学部附属病院 臨床試験部 データマネジメント室

TEL：043-222-1206　　　　FAX：043-222-1207

## 5.4. 登録されなかった被験者の取り扱い

登録において、不適格等の何らかの理由で登録が行われなかった被験者は、治験の登録症例には含めない。治験責任医師又は治験分担医師は、当該被験者に本治験への登録が不可である旨を説明する。

## 5.5. 個々の症例の中止基準

以下の基準に該当した場合、治験責任医師又は治験分担医師は治験機器施行を中止する。検査スケジュール記載の中止時検査・評価を実施する。

1. 被験者から治験参加の辞退の申し出や同意の撤回があった場合。
2. 治験登録後、被験者が対象として不適格であることが判明した場合。
3. 合併症の増悪により治験の継続が困難な場合。
4. 有害事象により治験の継続が困難な場合。
5. 妊娠が判明した場合。
6. 治験全体が中止された場合。
7. その他の重大な治験実施計画書違反が判明した場合。
8. 治験責任医師又は治験分担医師の判断により中止の必要性が認められた場合。

- 治験責任医師又は治験分担医師は中止日、中止理由等の必要事項を記録する。また、中止時の規定項目を可能な範囲で行う。
- 2), 7) 以外は治験が中止された場合の「中止日」は、中止の理由となる事象が発現した日又は判明した日ではなく、治験責任医師又は治験分担医師が中止を判断した日とする。
- 治験責任医師又は治験分担医師は、治験機器施行開始後に中止基準に該当する被験者が判明した場合には、当該被験者にその旨を説明して治験を中止し、適切な処置を行う。
- 有害事象（臨床検査値の異常変動を含む）により中止した場合、退院又は転院が可能と判断できるまで、必要な検査・観察を実施し、治験機器との因果関係が否定出来ない有害事象については、原則として症状が回復又は軽快するまで可能な限り観察継続する。

## 5.6. 併用可能薬及び併用可能療法

併用薬は指定しない。併用療法については、評価眼に対する眼科手術を行う必要が生じた際には、当該被験者の治験を中止とする。

## 5.7. 併用禁止薬及び併用禁止療法

- 他の治験薬、治験製品及び治験機器
- エタンブトール塩酸塩錠及び／あるいはアミオダロン塩酸塩錠

## 5.8. 併用制限薬

前観察期間（スクリーニング）前28日以内から治験終了まで、以下の用量・用法を変更しない。

- 内服ステロイド製剤（頓用を除く）

　治験機器による治療開始前から使用している原疾患に対する併用薬は、用量・用法は変更しない。併用療法については、治験開始前から変更なく継続することは可能。

## 5.9. 治験中止後の対応

治験を中止した被験者には、退院又は転院が可能と医学的に判断できるまで、必要な検査・観察を実施し、必要に応じて適切な処置を講じる。

# 6. 治験機器

## 6.1. 外観及び構成

電気刺激装置、皮膚電極（エールローデ^®^、積水化成品工業㈱）及びこれらを接続する電極接続ケーブルで構成されている。

1. 本体

| 2. 電極パッド(エールローデ^®^) | 3. 電極リード線 |
| --- | --- |
| 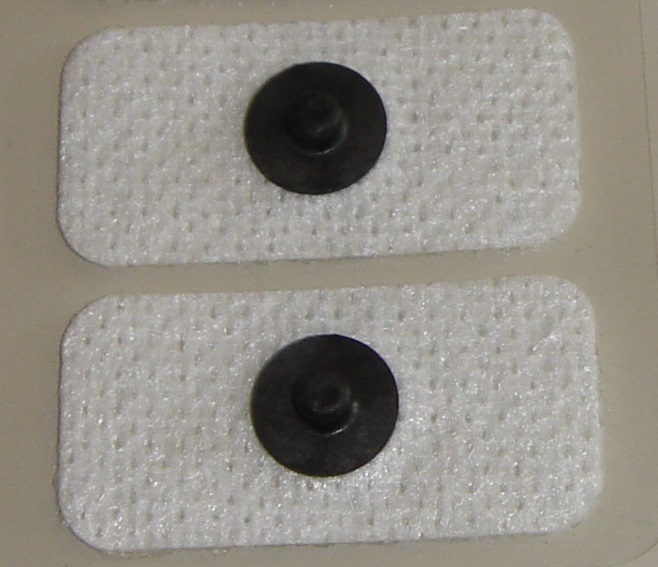 | 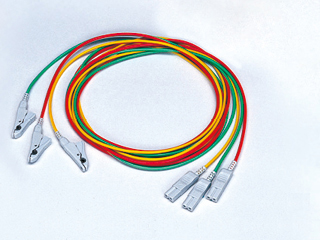 |

## 6.2 安全装置

装置には、非常停止ボタンを装備している。

機器は、ACアダプタから電源が供給される。ACアダプタは商用交流電源（100 VAC, 50/60 Hz）から直流5 Vの電源（DC 5 V）が生成される。ACアダプタはスイッチング方式で交流から所定の直流が生成される。スイッチング方式は、以下の過程を経て直流が生成される。

1. 100 VACをダイオードブリッジ整流器で正側に波が整流されコンデンサで平滑化される。
2. 高周波トランスで1次側が高速でon/offスイッチングされた電流が2次側に出力される。
3. 整流ダイオードを経て平滑化されて直流電源が出力される。

## 6.3. 作動原理

スイッチング方式は、回路構成が複雑であるが高耐圧部品により構成されている。電気的安全性試験で求められる耐圧試験にはスイッチング方式の方が性能的に優位であり、本装置で使用されているACアダプタもスイッチング方式によるものである。

CPU回路では、6個の設定を行うボタンスイッチ及び非常停止ボタンが押されたかどうかの状況の有無からの入力と、ダイヤルが時計回り／反時計回りに回転された状態の入力から、CPUに書き込まれたファームウェアにより、プログラムが実行される。プログラムの実行状況やボタンスイッチ及びダイヤルの状況は16文字×2行の液晶ディスプレイに表示される。CPUは20 MHzの水晶発振器によって正確なクロックで制御されている。

通電する電流は、PNPトランジスタ及びNPNトランジスタで定電流回路と8ビット（256段階）のデジタルポテンショメータ（デジタル可変抵抗器）によって電流量が正確に制御される。

正負両極刺激は論理回路によりOn/Offの情報がフォトカプラに入力され、光絶縁されて患者に電極が接続されている3個のソケットに出力される。

電気刺激を行うための電源は、直流5 Vを高速でon/offを実施してパルス状にトランスへ入力し、トランス出力で出力されたパルス状波形を平滑化して直流100 Vが生成される。

## 6.4. 品質

有限会社メイヨー稲沢研究所は、当局が定めたQMS省令に適合している事業所であると認定されており、医療機器登録製造所の認可を受けている。また、認証機関である株式会社コスモス・コーポレイション（認証機関番号：AG）により、認証を受けたすべての医療機器に対して、QMS定期調査により適合を認定されている。治験機器に対してはISO14971に従ってリスクマネジメントを実施し、当該治験機器においても品質は担保されている。

### 6.4.1. 安全性

当該治験機器は、以下の電気的安全性に関する医療機器のJIS規格に適合するように設計されている。

| 規格番号 | 標題 |
| --- | --- |
| JIS T 0601-1 | 医用電気機器−第 1 部：基礎安全及び基本性能に関する一般要求事項 |
| JIS T 0601-1-2 | 医用電気機器−第 1-2 部：安全に関する一般的要求事項−電磁両立性−要求事項及び試験 |
| JIS T 0601-2-10 | 医用電気機器−第 2-10 部：神経及び筋刺激装置の安全に関する個別要求事項 |

　基本的な電気的安全性試験とされている耐電圧試験、漏れ電流試験、低抵抗試験及び消費電力については、規格の要求事項に適合しており患者の安全性を担保すると考える。安全性試験においては量産機について実施予定である。本治験時には、JIS T0601-1及びJIS T0601-1-2に適合している付属のACアダプタを使用し、電磁的影響に関し他の電子機器から電磁的な影響を受けない、また与えないような空間距離を担保する等安全対策を講じたうえで実施する。

使用する電流量からは有害事象が発生する可能性は低いと推測されるが、電極装着部位に一致した皮膚炎や角膜障害が生じる可能性がある。また電気刺激による電極装着部位付近の皮膚に刺激感を感じることが予測される。

### 6.4.2. 性能

　医療機器のJIS規格 T0601-2-10で要求される精度を含めて以下に表示する。

- 電流値：　　0~±2,000 μA（精度 定格出力0~±30 %）
- 刺激頻度：　20 Hz（精度±1 %）
- 出力波形：　正負両相
- パルス幅：　各相5~10 msec（精度±1 %）

### 6.4.3. 製造

材料、部品、構成品

当該品目に係る品質管理システムの範囲は太線で囲んだ箇所である。

材料、部品、構成品の受入検査

製造、加工ならびに工程内検査

点線で囲った範囲は、下記製造所の工程範囲である。

製造所名称：

　有限会社メイヨー　稲沢研究所

製造所所在地：

　愛知県稲沢市高御堂二丁目25番22号

製造業許可番号：　23BZ005022

製造業許可区分：　一般

製品検査

包装・表示

保管、出荷可否決定

出荷

## 6.5. 包装・表示

　＜治験機器ラベル（見本）＞


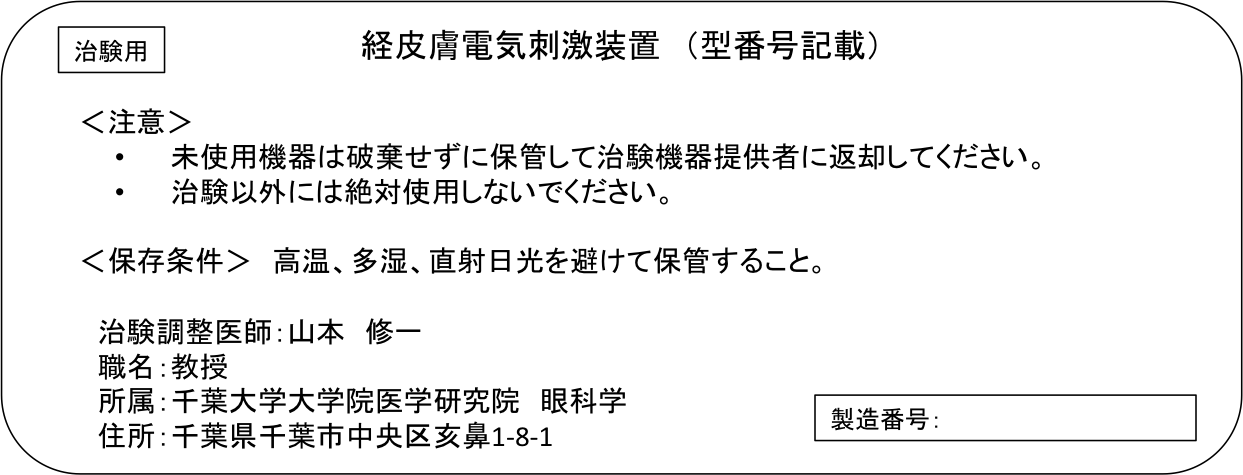


ラベルには治験調整医師の職名及び経皮膚電気刺激装置の製造番号を記載する。

## 6.6. 管理方法

- 1. 治験調整医師は治験開始後速やかに治験機器を実施医療機関の治験責任医師へ交付する。
  2. 治験実施医療機関の治験機器管理者は、治験責任医師から実施医療機関の長を通じて提供された手順書に従って、治験機器を室温にて適切に管理する。
  3. 治験責任医師は、治験機器の供給される保存条件、使用期限等取扱い方法を説明した文書を作成し、これを実施医療機関の長、治験分担医師、治験協力者、治験機器管理者等に交付する。
  4. 治験責任医師及び治験分担医師は、治療を行う機器番号を確認し、治療中以外も含め治療機器に不具合が生じた場合は、その機器番号と不具合の状況を報告し、交換・修理が必要な場合は速やかに対応する。

# 7. 観察・検査・評価項目、方法及び実施時期

## 7.1 実施スケジュールと手順

観察・検査・評価の実施スケジュールを以下の表に示す。治験責任医師又は治験分担医師は、スケジュールに従って観察・検査等を実施する。なお、被験者背景の調査や臨床検査等、治験協力者が実施可能な項目については、治験責任医師の管理下で治験協力者が実施しても良い。（下記スケジュール表参照）

| 時期  項目 | 前観察期間 | 治療期間 | | | | | | | 中止時 |
| --- | --- | --- | --- | --- | --- | --- | --- | --- | --- |
|  | スクリーニング | 0週 | 2週 | 4週 | 6週 | 8週 | 10週 | 12週 |  |
| 来院治療 | 来院1〜2 | 来院3 | 来院4 | 来院5 | 来院6 | 来院7 | 来院8 | 来院9 |  |
| 許容範囲（日） | 0～28日 | 0 | ±4 | ±4 | ±4 | ±4 | ±4 | ±4 | ±7 |
| 同意取得 | ● |  |  |  |  |  |  |  |  |
| 登録^a^ |  | ● |  |  |  |  |  |  |  |
| 患者背景の確認 | ● |  |  |  |  |  |  |  |  |
| 経皮膚電気刺激治療（TdES） |  | ● | ● | ● | ● | ● | ● |  |  |
| 自他覚症状^b^ | ● | ● | ● | ● | ● | ● | ● | ● | ● |
| 血圧^c^ | ● | 〇 | 〇 | 〇 | 〇 | 〇 | 〇 | 〇 | 〇 |
| 血液生化学的検査（採血）^d^ | ● |  |  |  |  |  |  |  |  |
| 造影MRI | ● |  |  |  |  |  |  |  |  |
| logMAR視力（矯正小数視力） | ● | ● | ● | ● | ● | ● | ● | ● | ● |
| ETDRS視力 | ● | ● |  |  |  |  |  | ● | ● |
| 静的視野検査^e^ | ● |  |  |  |  |  |  | ● | ● |
| 細隙灯顕微鏡検査 | ● |  |  |  | ● |  |  | ● | ● |
| 眼圧・眼底検査 | ● |  |  |  | ● |  |  | ● | ● |
| 妊娠検査^f^ | ● |  |  |  |  |  |  |  | 〇 |
| 有害事象の観察^g^ |  |  |  |  |  |  |  |  |  |

1. 同意取得と登録までの期間が28日間を超えた場合は、再同意を取得し登録可能とする。
2. 自他覚症状（角膜炎、皮膚炎の他、前眼部・中間透光体・眼底の評価、顔面神経や三叉神経、鼻への影響も確認）はTdES実施前と実施直後に実施する。
3. 血圧は、スクリーニング時に測定し、それ以外は必要に応じて測定する。
4. 臨床検査項目は、ALT(GOT)、AST(GPT)、ALP、総ビリルビン、BUN、血清クレアチニン、HbA1c、赤血球、白血球、ヘモグロビン、血小板、ESR、CRPとする。
5. 前観察期間の静的視野検査（HFA）10-2プログラムは、計測値の信頼性（固視不良率20 %未満、偽陽性率15 %未満、偽陰性率33 %未満）を確認する。信頼性が確認できなかった場合は再測定するか、発症後6ヶ月以内の自施設での計測値を使用可とする（他施設での計測値は使用不可）。静的視野検査（HFA）は10-2プログラムとエターマンテスト（100点）を実施する。
6. スクリーニング時、女性被験者を対象に、実施医療機関で定められた基準に従い、尿中hCG検査を実施する。ただし、両側卵巣摘出又は子宮摘出の既往歴がある被験者、又は別の医学的理由を伴わずに月経のない状態が12ヶ月以上にわたる被験者は実施不要とする。中止時は必要に応じてスクリーニング時に実施した被験者を対象として実施する。
7. 有害事象とは副作用等好ましくないすべての事象のことで、治療との因果関係は問わない。

### 7.1.1. スクリーニング検査

同意取得後、スクリーニング検査を開始する。治験責任医師又は治験分担医師は以下のスクリーニング検査を行い、選択基準を満たし、除外基準に抵触しない患者を登録する。検査項目は以下に記載のとおりとする。

1. 自他覚症状

※角膜炎、皮膚炎の他、前眼部・中間透光体・眼底の評価、顔面神経や三叉神経、鼻への影響も確認する。

1. 血圧
2. 血液生化学的検査（採血）

※ALT(GOT)、AST(GPT)、ALP、総ビリルビン、BUN、血清クレアチニン、CRP 、HbA1c、ESR、赤血球、白血球、ヘモグロビン、血小板数を測定する。

1. 造影MRI
2. logMAR視力（矯正小数視力）
3. ETDRS視力
4. 静的視野検査（HFA）：10-2プログラム及びエスターマンテスト（100点）
5. 細隙灯顕微鏡検査
6. 眼圧（非接触眼底測定）・眼底検査（カラー眼底写真）
7. 妊娠検査

※スクリーニング時、女性被験者を対象に、実施医療機関で定められた基準に従い、尿中hCG検査を実施する。ただし、両側卵巣摘出又は子宮摘出の既往歴がある被験者、又は別の医学的理由を伴わずに月経のない状態が12ヶ月以上にわたる被験者は実施不要とする。

1. 有害事象の観察

なお、4)及び7) に関しては、同意取得前のデータを使用することができる。

### 7.1.2. 被験者の情報

同意取得時又はスクリーニング検査時に、以下の被験者情報を記録する。

1. 同意取得日
2. 被験者識別コード
3. 性別
4. 年齢
5. 身長・体重
6. 確定診断時の造影MRI所見
7. 既往・合併症
8. 診断名
9. 前治療歴
10. 治験期間中に使用する予定の薬剤に対するアレルギーの有無

### 7.1.3. 観察・検査・評価項目

各来院時における検査項目を以下に記す。なお、治験責任医師又は治験担当医師の判断で、安全性上等の必要に応じて検査を追加することは可能とする。

#### 7.1.3.1. 0週

- 治験機器施行前に以下を実施する。

1. 自他覚症状

※角膜炎、皮膚炎の他、前眼部・中間透光体・眼底の評価、顔面神経や三叉神経、鼻への影響も確認する。

1. logMAR視力（矯正小数視力）
2. ETDRS視力
3. 有害事象の観察

- 治験機器施行直後に以下を実施する。

1. 自他覚症状

　　※角膜炎、皮膚炎の他、前眼部・中間透光体・眼底の評価、顔面神経や三叉神経、鼻への影響も確認する。

1. 有害事象の観察

#### 7.1.3.2. 2, 4, 8, 10週

- 治験機器施行前に以下を実施する。

1. 自他覚症状

　　※角膜炎、皮膚炎の他、前眼部・中間透光体・眼底の評価、顔面神経や三叉神経、鼻への影響も確認する。

1. logMAR視力（矯正小数視力）
2. 有害事象の観察

- 治験機器施行直後に以下を実施する。

1. 自他覚症状

　　※角膜炎、皮膚炎の他、前眼部・中間透光体・眼底の評価、顔面神経や三叉神経、鼻への影響も確認する。

1. 有害事象の観察

#### 7.1.3.3. 6週

- 治験機器施行前に以下を実施する。

1. 自他覚症状

　　※角膜炎、皮膚炎の他、前眼部・中間透光体・眼底の評価、顔面神経や三叉神経、鼻への影響も確認する。

1. logMAR視力（矯正小数視力）
2. 細隙灯顕微鏡検査
3. 眼圧（非接触眼底測定）・眼底検査（カラー眼底写真）
4. 有害事象の観察

- 治験機器施行直後に以下を実施する。

1. 自他覚症状

　　※角膜炎、皮膚炎の他、前眼部・中間透光体・眼底の評価、顔面神経や三叉神経、鼻への影響も確認する。

1. 有害事象の観察

#### 7.1.3.4. 12週

- 来院時に以下を実施する。

1. 自他覚症状

　　※角膜炎、皮膚炎の他、前眼部・中間透光体・眼底の評価、顔面神経や三叉神経、鼻への影響も確認する。

1. logMAR視力（矯正小数視力）
2. ETDRS視力
3. 静的視野検査（HFA）：10-2プログラム及びエスターマンテスト（100点）
4. 細隙灯顕微鏡検査
5. 眼圧（非接触眼底測定）・眼底検査（カラー眼底写真）
6. 有害事象の観察

#### 7.1.3.5. 中止時

治験責任医師又は治験分担医師により治験機器施行の中止を判断された日から7日以内に可能な範囲で以下を実施する。

1. 自他覚症状

　　※角膜炎、皮膚炎の他、前眼部・中間透光体・眼底の評価、顔面神経や三叉神経、鼻への影響も確認する。

1. logMAR視力（矯正小数視力）
2. ETDRS視力
3. 静的視野検査（HFA）：10-2プログラム及びエスターマンテスト（100点）
4. 細隙灯顕微鏡検査
5. 眼圧（非接触眼底測定）・眼底検査（カラー眼底写真）
6. 有害事象の観察
7. 妊娠検査（必要に応じてスクリーニング時に実施した被験者を対象として実施する。）

# 8. 有害事象発生時の取り扱い

## 8.1. 有害事象、不具合及び機器関連有害事象の定義

有害事象とは、治験機器との因果関係の有無に関わらず、当該治験機器の使用時に被験者、使用者その他の者に生じたすべての好ましくない又は意図しない疾病又は障害並びにその兆候（臨床検査値の異常を含む。）をいう。ただし、被験者以外の者に生じたものについては、治験機器の使用による影響と疑われるものに限る。

既存の病態（治験期間よりも以前に存在した既往歴又は合併症）については、治験期間内に増悪又は発症頻度の上昇がない限り、有害事象として扱わない。ただし、治験機器施行後に合併症が悪化した場合、有害事象として取扱い、悪化が確認された日を有害事象の発現日とする。

不具合とは、破損、作動不良等広く品質、安全性、性能等に関する治験機器の具合の良くないことをいい、設計、交付、保管、使用のいずれの段階によるものであるかを問わない。治験機器の不具合に関する情報を以下、不具合情報という。

機器関連有害事象とは、有害事象のうち本治験機器又は手技との因果関係が否定できないものをいう。

## 8.2. 有害事象又は不具合発生時の被験者への対応

治験責任医師又は治験分担医師は、有害事象又は不具合を認めたときは、直ちに適切な処置を行うとともに、治験機器の使用を中止した場合や、有害事象に対する治療が必要となった場合には、被験者にその旨を伝える。なお、治験終了・中止時に治験機器との因果関係が否定できない有害事象が未回復の場合は、原則として回復又は軽快するまで可能な限り観察を継続する。ただし、治験責任医師又は治験分担医師が本治験の影響は消失しており、被験者の安全性は十分確保され、それ以上の追跡調査は必要ないと判断した場合はこの限りではない。

## 8.3. 報告の対象となる有害事象及び不具合

治験機器が施行されてから治験終了時までに発生したすべての有害事象は、治験機器との因果関係の有無に関わらず報告し、有害事象が消失するか治験期間終了時（中止時）4週まで観察する。また、治験機器との因果関係が否定できないと判断された有害事象及び発生した不具合情報については治験期間終了時まですべて報告する。

## 8.4. 有害事象発生時の報告手順

上記期間に発生したすべての有害事象及び不具合について、治験責任医師又は治験分担医師は、カルテならびに症例報告書に齟齬なく記載する。

## 8.5. 有害事象の評価に必要な記載内容

### 8.5.1. 有害事象

1. 有害事象の名称

有害事象名は、原則として診断名・疾患名（病名）で記録する。診断名・疾患名が特定できない場合や治験責任医師又は治験分担医師より診断名・疾患名としないことが妥当と判断された場合には、臨床症状又は徴候を有害事象名とする。

1. 発現日
2. 消失日：回復、回復又は消失したが後遺症あり、死亡の場合に日付
3. 転帰：回復、軽快、回復又は消失したが後遺症あり、未回復、死亡、不明
4. 処置（治験機器の施行）：変更なし、中止、該当せず
5. その他処置：なし、薬物治療、その他
6. 重篤度：非重篤、重篤
7. 重症度：軽度、中等度、重度

- 軽　度：日常生活に支障を来たさないと考えられる程度の有害事象。 例）経過観察可能な程度
- 中等度：日常生活に支障を来たすと考えられる程度の有害事象。 例）何らかの介入や治療が必要な程度
- 重　度：日常生活を困難にすると考えられる程度の有害事象。 例）高度かつ集中的な治療や全身介助が必要とされる程度

1. 治験機器との因果関係：関連が否定できる、関連が否定できない

### 8.5.2. 不具合

1. 不具合名
2. 不具合の確認日
3. 発生日
4. 不具合が発生したと考えられる原因及び不具合状況
5. 不具合に対する処置
6. 当該不具合による有害事象の有無

### 8.5.3. 有害事象の回復性と治験機器との因果関係

#### 8.5.3.1. 治験機器との因果関係

有害事象の回復とは、有害事象がない状態、又は治験機器施行前の状態への回復とする。有害事象における治験機器との因果関係の判定に際しては、被験者の全身状態、合併症、併用薬・併用療法、時間的関係を勘案して判断する。

- 関連が否定できる：被験者が治験機器施行開始前、あるいは以下の事例に示すように、他の要因によるものであると説明できる場合

1. 当該有害事象が併用薬の既知の副作用である場合
2. 治験機器を再施行しても同一の有害事象が発現しない

- 関連が否定できない：他の要因との関連がはっきりできない場合

#### 8.5.3.2. 治験機器に関する処置

1. 変更なし：有害事象が発現したが、治験機器治療の施行条件に変更がない場合
2. 中止：有害事象の発現により、治験機器施行を中止した場合
3. 減量：有害事象の発現により、治験機器の電流強度又は施行時間を減量した場合
4. 休止：有害事象の発現により、治験機器施行を一時的に休止した場合
5. 増強：有害事象の発現により、治験機器の電流強度を増強又は施行時間を延長した場合
6. 該当せず：有害事象が治験機器施行開始前又は治験期間終了後に発現した場合

#### 8.5.3.3. 転帰

1. 回復：有害事象が発現以前の状態に回復、消失した場合
2. 回復したが後遺症あり：有害事象が回復したが、有害事象の影響が後遺症として残っている場合
3. 死亡：発現した有害事象が死亡の直接の原因となった場合
4. 軽快：有害事象は継続しているが、症状は改善傾向である場合
5. 未回復：有害事象は継続している（症状は改善傾向でない）場合
6. 不明：被験者の追跡が不能となった場合

## 8.6. 重篤な有害事象発生時の取り扱い

### 8.6.1. 重篤な有害事象の定義

重篤な有害事象とは、次のいずれかに該当するものとする。

1. 死亡に至るもの（死亡）
2. 生命を脅かすもの（死亡につながるおそれ）
3. 入院又は入院期間の延長が必要になるもの
4. 永続的又は顕著な障害・機能不全に陥るもの
5. 先天性異常をきたすもの
6. その他、上記に準じて重篤なもの

なお、以下に記載する理由での「入院」については重篤な有害事象とはみなさない。

- 再検査、追跡調査のための入院又は入院期間の延長、及び治験開始前より予定していた治療又は検査を治験中に実施することのみを目的とした入院（予定手術や検査等）は含まれない（ただし、その入院中新たに発生したものは有害事象として取り扱う）。
- 本治験の対象疾患に関係のない既存の病態に対する待機的治療のための入院。
- 全身状態の悪化を伴わない、社会的事由及び介護者の一時休息のための入院。

### 8.6.2. 報告の対象となる重篤な有害事象

報告対象は、スクリーニング開始時から治験終了（中止）時までに発生した、すべての重篤な有害事象又はその発生のおそれがある事象、及び治験終了（中止）後に治験機器との関連性が疑われる重篤な有害事象とする。

### 8.6.3. 重篤な有害事象の報告手順

有害事象が発生し、治験責任医師等が重篤と判断した場合、次の手順に従い当該有害事象情報を取り扱う。

1. 治験責任医師から実施医療機関の長への報告

治験責任医師は、治験機器施行後、治験期間中に治験機器の不具合等による死亡その他の重篤な有害事象の発生を認めた場合又はその発生のおそれがあると認めた場合は、直ちに各実施医療機関の長に文書にてその内容を報告する。報告に際しては、重篤な有害事象が予測出来ないか否かを特定する。

また、治験責任医師は可能な限り速やかに「重篤な有害事象及び不具合に関する報告書」（統一書式14）及び「医詳細記載用書式」に当該事象の詳細な内容を記載し、実施医療機関の長に報告する。

1. 治験責任医師から治験調整医師、治験製品提供者への報告

治験責任医師は、治験機器施行後、治験期間中に治験機器の不具合等による死亡その他の重篤な有害事象の発生を認めた場合又はその発生のおそれがあると認めた場合は、直ちに治験調整医師及び治験機器提供者にその内容を報告する。

また、治験責任医師は可能な限り速やかに「重篤な有害事象及び不具合に関する報告書」（統一書式14_及び医詳細記載用_書式12_14_19共通）に当該事象の詳細な内容を記載し、治験調整医師に報告する。

1. 治験責任医師と治験調整医師との協議

治験責任医師は、必要に応じ治験調整医師と協議し、治験責任医師としての意見（厚生労働大臣への報告の必要性を含む。）を治験調整委員会に報告する。

また、効果安全性評価委員会に治験責任医師の判断について諮問を行った場合には、効果安全性評価委員会の意見に従う。

1. 厚生労働大臣への報告

薬機法に規定される報告対象となる有害事象又は不具合と判断した場合は、治験調整医師は「医療機器不具合・感染症症例報告書」（別紙様式第8）を作成し、効果安全性評価委員会及びPMDAへ報告する。

詳細な手順は「安全性情報の取り扱いに関する手順書（仮称）」に従う。

1. 実施医療機関の長への報告

治験責任医師は、厚生労働大臣への報告がなされた場合には、治験調整医師より入手した「医療機器不具合・感染症症例報告書」（別紙様式第8）及び必要に応じ「医詳細記載用書式」の内容を、可能な限り速やかに実施医療機関の長に報告する。

1. 追加情報の入手時の対応

当該有害事象が発生した実施医療機関の治験責任医師は、当該事象に関する追加情報が得られた場合には、可能な限り速やかに各実施医療機関の長に追加報告を行うとともに、治験調整医師及び治験機器提供者に報告する。当該追加情報の取扱いは、上記1)~5)の手順に準ずることとし必要に応じPMDAへの報告等を行う。

# 9. 評価項目

## 9.1. 主要評価項目

0週時との比較による12週時におけるlogMAR視力（矯正小数視力から換算）。

【主要評価項目の設定根拠】

logMAR視力：国内で実施される視力の維持・改善効果を検討する臨床研究に広く用いられているため。

## 9.2. 副次評価項目

1. logMAR視力の0週からの変化量。
2. ETDRSチャートを用いたETDRS視力の0週から12週までの変化量。
3. 静的視野検査（HFA）：10-2プログラム MD値の網膜感度のベースラインから12週までの変化量。
4. 静的視野検査（HFA）：エスターマンテスト（100点）スコアベースラインから12週までの変化量。

【副次評価項目の設定根拠】

- ETDRS視力：欧米及び本邦で広く使用されている視力評価であり視機能判定として臨床現場で定着しているため。
- 静的視野検査（HFA）10-2プログラム MD値：中心約10度部分以内の視野を詳しく測定するのに向いているため。
- 静的視野検査（HFA）エスターマンテスト（100点）MD値：周辺視野を評価する方法として一般的なゴールドマン動的視野は、検者によるばらつきが大きく、また結果を数値化する際にもばらつきが生じる。そのため静的視野検査での周辺視野評価法であるエスターマン視野での評価を探索的に行うこととした。

## 9.3. 安全性評価項目

12週間における有害事象の発現状況（種類、頻度及び重症度等）

被験者に観察された有害事象をすべて記録し有害事象の発現状況（種類、頻度及び重症度等）を解析する。第Ⅱ相試験では有害事象の発現が見られなかったが、角膜炎、皮膚炎の他、前眼部・中間透光体・眼底の評価も行う。また顔面神経や三叉神経、鼻への影響も受診の際に確認を行う。

【安全性評価項目の設定根拠】

本治験の安全性について評価するため設定した。

# 10. 統計学的事項

本治験の統計解析計画の概要を以下にまとめた。なお、統計解析計画の詳細は、統計解析計画書に記載する。統計解析計画書において本治験実施計画書の概要を修正することがあるが、主要評価項目の定義や解析方法が変更される場合には、本治験実施計画書を改訂する。

## 10.1. 解析対象集団

### 10.1.1. 安全性解析対象集団

本治験に登録され、少なくとも1回は治験機器が施行された症例を安全性解析対象集団とする。ただし、医療機器GCP省令不遵守例は安全性解析対象集団から除外する。

### 10.1.2. 最大の解析対象集団

本治験に登録され、1回以上治験機器が施行され、有効性データがあるすべての被験者を最大の解析対象集団（以下、「FAS」という。）とする。ただし、ベースラインのデータが取得されなかった被験者及び、重大な治験実施計画書違反 （同意未取得、等）の被験者については除外する。

### 10.1.3. 治験実施計画書に適合した対象集団

FASから、治験方法や併用療法等、治験実施計画書の規定に対して、以下の重大な違反があった症例を除いた被験者集団（以下、「PPS」という。）とする。

選択基準違反、除外基準違反、併用禁止薬違反、併用禁止療法違反、80 %未満の治療率

## 10.2. 目標症例数と設定根拠

主要評価項目解析対象例数：　　　5例5眼

副次評価項目解析対象例数：　　　5例5眼

安全性評価：　　　　　　　　　　5例

【設定根拠】

NAIONは希少疾患であることから、1年間で組み入れ可能な症例数とした。

## 10.3. 症例の取り扱い

原則として登録された症例については、治験調整医師及び統計解析責任者が協議の上、症例の取り扱いを決定する。新たな問題が起こった場合の症例の取り扱いについても、治験調整医師及び統計解析責任者が、協議の上、決定する。

## 10.4. データの取り扱い

データ集計・解析時におけるデータの取り扱いについては、原則として以下に示すとおりとする。疑義が生じた場合は、統計専門家と治験調整医師が協議の上データ固定前に決定する。

欠測値に対しては補完を行わない。

## 10.5. 統計解析項目及び解析計画

すべての症例において治験機器の使用が終了し、データが固定された後に解析を行う。

有効性評価において、FAS及び PPSにおける解析を行う。FASとPPSが同じとみなせる集団の時はPPSの解析は実施しない。

統計解析の詳細はデータ固定前に別途作成する統計解析計画書に規定する。

＜主要評価項目＞

- 0週時との比較による12週時におけるlogMAR視力（矯正小数視力から換算）。

＜副次評価項目＞

- logMAR視力
- ETDRS視力
- 静的視野検査（HFA）：10-2プログラム
- 静的視野検査（HFA）：エスターマンテスト（100点）

＜安全性評価項目＞

- 12週間における有害事象の発現状況（種類、頻度及び重症度等）

### 10.5.1. 被験者背景の解析

各解析対象集団における被験者背景データの分布及び要約統計量を算出する。名義変数については、カテゴリの頻度及び割合を示す。連続変数については要約統計量を算出する。

### 10.5.2. 安全性及び有効性の解析

#### 10.5.2.1. 主たる解析

視力（矯正小数視力をlogMAR視力に換算したスケールを用いて、12週目のベースラインからの変化量を解析する。統計手法としては線形混合効果モデルを用い、仮説検定の有意水準は両側5 %とし、信頼区間は両側95 %信頼区間を算出する）。なお、モデル解析が実施できない場合には、要約統計量のみを算出する。詳細な解析手法に関しては、統計解析計画書に記載をする。

#### 10.5.2.2. 副次解析

有効性の副次評価項目の解析を行う。視力（矯正小数視力をlogMAR視力に換算したもの、及びETDRS視力）、静的視野検査（HFA）10-2プログラム及びエスターマンテスト（100点）についての統計解析を行う。有効性の副次評価項目の解析では多重性の調整は行わない。統計手法としては線形混合効果モデルを用い、仮説検定の有意水準は両側5 %とし、信頼区間は両側95 %信頼区間を算出する。なお、モデル解析が実施できない場合には、要約統計量のみを算出する。詳細な解析手法に関しては、統計解析計画書に記載をする。

### 10.5.3. 安全性の解析

安全性の解析は、有害事象を集計し、発現の有無及び重篤度について、発現例数及び割合を集計し、一覧表を作成する。また、発現の有無に対する 2項分布の正確な両側 95 % 信頼区間を算出し、必要に応じ適切な有意差検定を実施する。

## 10.6. 効果安全性評価委員会

本治験では効果安全性評価委員会を設置する。効果安全性評価委員会は治験責任医師と独立した機関として設立され、本治験とは独立した立場である 3人以上の専門家による委員で構成される。効果安全性評価委員会は、被験者の安全性を確保することを目的に、必要に応じて、被験治療における有害事象発現率の比較、重篤な有害事象に関する詳細な検討等の安全性モニタリングを行う。ときにその結果を踏まえて有害事象のリスクを軽減する為に、組入れ基準の変更等の治験デザインの変更を勧告すること、あるいは治験の継続の可否を判断することもある。詳細は「効果安全性評価委員会に関する標準業務手順書」に従う。

## 10.7. 最終解析

追跡期間終了後、データが得られ症例が固定された後に解析を行う。統計解析責任者が「解析報告書」をまとめ、治験調整医師及び治験責任医師に提出する。治験調整医師は解析報告書の内容を総括し、治験全体の結論、問題点、結果の解釈及び考察、今後の方針等を主として臨床的観点からまとめた「総括報告書」を作成し、治験責任医師の承認を得る。

# 11. 治験実施計画書の遵守及び逸脱

1. 治験責任医師又は治験分担医師は、本治験実施計画書を遵守して治験を実施する。
2. 治験責任医師又は治験分担医師は、治験実施計画書から逸脱した場合、すべての逸脱の詳細及び理由を記録する。
3. 被験者の緊急の危機を回避するためその他医療上やむを得ない理由により実施計画書から逸脱した場合、治験責任医師は、逸脱の内容及びその理由を記載した文書を実施医療機関の長に直ちに提出するとともに、当該文書の内容を実施医療機関の長を経由して治験審査委員会に速やかに報告する。

# 12. 治験実施計画書、症例報告書又は解析計画に関する変更

## 12.1. 治験実施計画書及び症例報告書の改訂

治験実施計画書及び症例報告書を改訂する場合には、以下の手順により行う。

1. 治験責任医師は、治験機器の品質、有効性及び安全性に関する事項、その他治験を適正に行うために重要な情報を知ったときは、必要に応じて当該治験実施計画書を改訂する。また、改訂の際には改訂履歴を作成し、それを保存する。
2. 治験責任医師は、必要に応じ治験実施計画書の改訂に併せて又は他の理由により症例報告書を改訂する。また、改訂の際には改訂履歴を作成し、それを保存する。
3. 治験責任医師は治験実施計画書改訂版及び症例報告書用紙改訂を速やかに実施医療機関の長に提出し、実施医療機関の長を経由して速やかに治験審査委員会の承認を得る。
4. 治験審査委員会の意見に基づく実施医療機関の長の指示が治験責任医師の許容できる範囲内で、治験実施計画書及び症例報告書用紙を修正する場合も同様の手順とする。

## 12.2. 統計解析計画の変更

統計解析責任者は、統計解析計画書の内容を変更した場合、変更内容をすべて本治験の統計解析報告書に記載する。なお、統計解析計画書の変更は、その経緯を記録に残す。

# 13. 治験の中止、中断又は終了

## 13.1. 治験全体での中止又は中断の基準

治験調整医師は、以下の情報が得られ、治験全体の続行が困難であると考えられる時には、治験責任医師と治験全体の中止又は中断について協議のうえ、決定する。

1. 治験機器に関する新たな安全性情報又は重篤な有害事象及び不具合情報が得られる等、治験の安全性確保が困難になった場合。
2. 実施医療機関が、重大な医療機器GCP省令違反、治験実施計画書からの重大な逸脱を行った場合。
3. その他、治験実施中に治験の中止・中断が必要と考えられる新たな情報が得られた場合。

## 13.2. 治験全体での中止又は中断する場合の手続き

治験調整医師は、他の治験責任医師との協議又は効果安全性委員会等への諮問により治験全体を中止又は中断する場合には、実施医療機関の長及びPMDAにその旨とその理由を詳細に速やかに文書で通知する。また、治験機器施行中の被験者に対して速やかにその旨を伝え、適切な治療への変更等の適切な処理を行うものとする。

## 13.3. 個々の医療機関での治験の中止又は中断する場合の手続き

治験責任医師は、治験を中止又は中断した場合には、実施医療機関の長に速やかにその旨を文書で通知するとともに、中止又は中断について文書で詳細に説明する。

治験調整医師は、治験責任医師が治験を中止又は中断した旨を通知してきた場合には、中止又は中断について詳細に説明された文書により当該治験に関与するすべての治験責任医師及びPMDAに速やかに文書で通知する。

## 13.4. 治験の終了

治験責任医師は、治験終了後、実施医療機関の長に治験が終了した旨を文書で通知し、治験結果の概要を文書で報告する。

# 14. データマネジメント

## 14.1. データマネジメントの手順

データマネジメントに関する詳細な手順については、データマネジメント計画書に記載する。

## 14.2. データの収集

治験責任医師又は治験分担医師は、21 CFR Part 11、医療機器GCP省令及びER/ES指針の要件に対応したElectronic Data Capture（以下、「EDC」という。）を用いた症例報告書を作成する。治験責任医師又は治験分担医師は、症例報告書の記載内容の変更、修正又は追記に当たっては、症例報告書を作成したEDC上で行い、すべて電子情報として記録する。なお、治験責任医師は、治験分担医師が症例報告書を作成した場合並びに治験協力者が原資料（原データ）から症例報告書に転記した場合には、当該症例報告書が提出される前にその内容について点検し、問題がないことを確認する。治験責任医師は、最終的に電子症例報告書を電子媒体（例：CD-R等）にて実施医療機関へ提供する。治験責任医師は、電子症例報告書の見読性、保存性を担保する。

EDCシステムの使用にあたり、実施医療機関はEDCトレーニングを受講し、入力方法の詳細は別途入力マニュアルを参照する。

## 14.3. 症例報告書に直接記入され、かつ原資料（原データ）と解すべき資料の特定

- 本治験においては、以下の文書等を原資料（原データ）とする。

1. 被験者の同意及び被験者への情報提供に関する記録、診療録、看護記録、臨床検査データ及び画像検査フィルム等症例報告書作成の基となった記録。なお、電子カルテに格納されたデータも原資料とみなす
2. 治験機器施行に関する記録
3. 本治験に関連する指針上必要な治験に係る文書又は記録

- 症例報告書に記載されたデータのうち、以下に示す項目は症例報告書の記載をもって原資料（原データ）とする。ただし、診療録等に記載のある場合は、当該診療録等を原資料（原データ）とみなす。

1. 併用薬・併用療法の目的
2. 有害事象の程度、転帰（追跡調査時の結果を含む）、重篤度、治験機器との因果関係の判定及び判定根拠
3. 被験者の治験中止理由
4. 治験責任医師又は治験分担医師のコメント

# 15. 記録の保存

## 15.1. 実施医療機関による記録の保存

医療機器GCP省令に規定される実施医療機関において保存すべき治験に係る文書又は記録は、病院長が次の日のうちいずれかの遅い日までの期間保存する。

1. 治験機器提供者が被験機器に係る医療機器についての製造販売の承認を受ける日から5年が経過した日（開発が中止された場合には、開発中止が決定された日から3年が経過した日）
2. 治験の中止又は終了後3年が経過した日

治験責任医師は、治験実施医療機関又は治験審査委員会が保存すべき記録について保存が不要となった場合、実施医療機関に通知する。

## 15.2. 治験責任医師による記録の保存

医療機器GCP省令に規定される治験責任医師が保存すべき治験に係る文書又は記録は、適切と判断される保管場所において、次のうちいずれか遅い日までの期間保存する。

1. 治験機器提供者が被験機器に係る医療機器についての製造販売の承認を受ける日から5年が経過した日（開発が中止された場合には開発中止が決定された日から3年が経過した日）。ただし、薬事法の規定により承認後の再審査を受けなければならない医療機器で、かつ再審査が終了するまでの期間が5年を超えるものについては、再審査が終了する日
2. 治験の中止又は終了後3年が経過した日

# 16. 原資料の直接閲覧

実施医療機関の長及び治験責任医師は、モニタリング、監査及び治験審査委員会又は規制当局による調査の際に、原資料等すべての記録を閲覧できることを保証する。治験が適切に実施されていること及びデータの信頼性が十分に確保されていることを確認する。直接閲覧の方法、実施時期についてはモニタリング計画書に別途定める。

# 17. 治験の品質管理及び品質保証

## 17.1. 品質管理

1. 本治験実施計画書からの逸脱した行為があった場合は、治験責任医師又は治験分担医師は、本実施計画書に定めるところに従う。
2. 治験責任医師又は治験分担医師は、症例報告書を本治験実施計画書に従って作成する。
3. 治験責任医師は、症例報告書に記載されたすべてのデータ及びその他の記録が正確及び完全であること確認する。
4. 症例報告書に記載されたデータのうち、原資料と何らかの矛盾がある場合は、治験責任医師はその理由を説明する記録を作成・保存する。
5. 治験調整医師は、当該モニタリングの対象となる実施医療機関において当該治験に従事していない者をモニターとして指名し、治験審査委員会の審査を経たモニタリング手順書に従いモニタリングを実施させる。モニターは、下記の事項を確認する。

　　－ 被験者の人権、安全及び福祉が保護されている。

－ 医療機器GCP省令、最新の治験実施計画書及び当該治験に係る手順書を遵守して実施している。

－ 治験責任医師又は治験分担医師から報告されたデータ等が正確かつ完全で原資料等の治験関連記録と照らし検証する。

1. データマネジメント責任者は、別途定めた標準業務手順書に従ってデータマネジメント計画を立案し、データの取扱い各段階で品質管理を行い、その品質を確保する。

## 17.2. 品質保証

　治験調整医師は、監査に関する計画書及び業務に関する手順書を作成し、治験審査委員会の意見を踏まえて、当該計画書及び手順書に従って監査を実施させる。なお、監査担当者は、当該監査に係る治験機器の開発を担当する者でなく、また、当該監査に係る実施医療機関において当該治験の実施（その準備及び管理を含む。）及びモニタリングに従事していない者とする。

# 18. 倫理及びGCP

本治験の実施に際しては、「ヘルシンキ宣言（2013年フォルタレザ総会改訂）」、薬機法及び医療機器GCP省令に従って行われる。また、本治験実施計画書及び当該治験に係る手順書を遵守して施行される。

また、治験責任医師又は治験分担医師は、被験者の選定にあたって、人権保護の観点ならびに選択基準と除外基準に基づいて被験者の健康状態、症状、年齢、性別、同意能力、治験責任医師等との依存関係、他の治験を含む臨床試験への参加の有無を十分に考慮したうえで、治験への参加を求めることの適否を慎重に検討する。

# 19. 治験審査委員会

本治験の実施に先立ち、実施医療機関の治験審査委員会は、本治験の倫理的、科学的及び医学的妥当性を審査する。本治験は、治験審査委員会の承認を得た後に実施する。治験審査委員会の審議結果が「修正の上で承認する」であった場合には、審議結果に基づいて実施計画書又は症例報告書、同意説明文書等を修正し承認した後、本治験を実施する。また、治験審査委員会は少なくとも1年に1回以上の頻度で本治験が適切に実施されているか否かを継続的に審査する。

# 20. 健康被害補償及び保険

本治験に参加した結果として被験者に健康被害が生じた場合、実施医療機関はその治療に関する医療体制の提供等必要かつ適切な処置を行う。ただし、本治験では自己負担分の医療費及び医療手当は支払われない。本治験に起因する健康被害により生じた賠償責任、補償責任への対応として、治験責任医師、治験分担医師、実施医療機関及び治験調整委員会等本治験に携わる者は医師主導治験保険（遺族補償金、葬祭料、障害補償金及び障害児補償金）に加入する。

# 21. 治験に関する費用負担

本治験に係わる費用は下記のとおりとする。

1. 本治験で使用される治験機器は有限会社メイヨーより提供される。
2. 治験機器施行期間中のすべての検査・画像診断等に係わる費用は保険外併用療養費を用いる。
3. 被験者の負担軽減費の支払いは、各実施医療機関が別途定める規程に従う。

# 22. 研究資金及び利益相反

本治験は、平成30年度千葉大学医学部附属病院先進医療開発推進経費（予定）にて実施する。有限会社メイヨーは、医療機器GCP省令上の治験機器に関する情報は提供するが、治験の実施、解析、報告に係わることはない。

本治験の利害関係については、各実施医療機関が別途定める規程に従い適切に審議する。

# 23. 治験のデータベース登録

本治験は、最初の被験者からの同意取得前にUMIN臨床試験登録システム（<http://www.umin.ac.jp/ctr/index-j.htm>）に登録を行う。

# 24. 治験実施体制

本治験の実施体制は、別紙1参照

# 25. 参考資料・文献リスト

1. 三宅養三，他: EER（Electrically　Evoked　Response）の臨床応用Ⅳ視神経疾患のEER解析. 日眼会誌 **84**: 2047-52, 1980.
2. Morimoto T, et al: Electrical stimulation enhances the survival of axotomized retinal ganglion cells in vivo. Neuro Report **13**: 227-30, 2002.
3. Okazaki Y, et al: Parameters of optic nerve electrical stimulation affecting neuroprotection of axotomized retinal ganglion cells in adult rats. Neurosci Res **61**: 129-35, 2008.
4. Morimoto T, et al. Transcorneal electrical stimulation rescues axotomized retinal ganglion cells by activating endogenous retinal IGF-1 system. Invest Ophthalmol Vis Sci. 46:2147-2155, 2005.
5. Sato T, et al: Direct effect of electrical stimulation on induction of brain-derived neurotrophic factor from cultured retinal Muller cells. Invest Ophthalmol Vis Sci. 49: 4641-4646, 2008.
6. Ni YQ, et al.: Neuroprotective effect of transcorneal electrical stimulation on light-induced photoreceptor degeneration. Exp Neurol. 219; 2009:439-452.
7. Ciavatta VT, et al.: Growth factor expression following implantation of microphotodiode arrays in RCS rats. Invest Ophthalmol Vis Sci. 47:3177, 2006.
8. Zhou WT, et al.: Electrical stimulation ameliorates light-induced photoreceptor degeneration in vitro via suppressing the proinflammatory effect of microglia and enhancing the neurotrophic potential of Muller cells. Exp Neurol. 238:192-208, 2012.
9. Wang X, et al.: Neuroprotective effect of transcorneal electrical stimulation on ischemic damage in the rat retina. Exp Eye Res. 93:753-760, 2011.
10. Kanamoto T, et al.: Proteomic study of retinal proteins associated with transcorneal electric stimulation in rats. J Ophthalmol. 1-6, 2015.
11. Fu L, et al.: Transcorneal electrical stimulation inhibits retinal microglial activation and enhances retinal ganglion cell survival after acute ocular hypertensive injury. Transl Vis Sci Technol. 7:1-11, 2018.
12. Yin H, et al.: Transcorneal electrical stimulation promotes survival of retinal ganglion cells after optic nerve transection in rats accompanied by reduced microglial activation and TNF-α expression. Brain Res. 1650: 10-20, 2016.
13. Morimoto T, Fukui T, Matsushita K, Okawa Y, Shimojyo H, Kusaka S, Tano Y, Fujikado T: Evaluation of residual retinal function by pupillary constrictions and phosphenes using transcorneal electrical stimulation in patients with retinal degeneration. Graefes Arch Clin Exp Ophthalmol. 244:1283-1292, 2006.
14. Fujikado T, et al: Effect of transcorneal electrical stimulation in patients with nonarteritic ischemic optic neuropathy or traumatic optic neuropathy. Jpn J Ophthalmol. 50:266-73, 2006.
15. Schatz A, et al. Transcorneal Electrical Stimulation for Patients With Retinitis Pigmentosa: A Prospective, Randomized, Sham-Controlled Follow-up Study Over 1 Year. Invest Ophthalmol Vis Sci. 58: 257-69, 2017.
16. Sato T et al. Effect of Electrical Stimulation on IGF-1 Transcription by L-Type Calcium Channels in Cultured Retinal Müller Cells. Jpn J Ophthalmol. 52: 217-23, 2008.
17. Willmann G, et al. Gene Expression profiling of the retina after transcorneal electrical stimulation in wild-type brown Norway rats. Invest. Ophthalmol Vis Sci. 52: 7529-37. 2011.
18. Schatz A, et al. Transcorneal electrical stimulation for patients with retinitis pigmentosa: a prospective, randomized, sham-controlled exploratory study. Ophthalmol Vis Sci. 52: 485-96, 2011.
19. Bittner AK, et al. Longevity of visual improvements following transcorneal electrical stimulation and efficacy of retreatment in three individuals with retinitis pigmentosa. Graefes Arch Clin Exp Ophthalmol. 256: 299-306. 2018.
20. Inomata K, et al. Transcorneal electrical stimulationof retina to treat longstanding retinal artery occlusion. Graefes Arch Clin Exp Ophthalmol 245: 1773-80, 2007.
21. Oono S, et al Transcorneal electrical stimulation improves visual function in eyes with branch retinal artery occlusion. Clin Ophthalmol 5: 397-402. 2011.
